# In Vitro Inhibition of Colon Cancer Stem Cells by Natural Polysaccharides Obtained from Wheat Cell Culture

**DOI:** 10.3390/polym17081048

**Published:** 2025-04-12

**Authors:** Alima Murtazina, Yaiza Jimenez-Martinez, Gloria Ruiz Alcala, Juan Antonio Marchal, Anel Tarabayeva, Elmira Bitanova, Izbasar Rakhimbayev, Gordon J. McDougall, Nazira Bishimbayeva, Houria Boulaiz

**Affiliations:** 1 Department of General Immunology, Faculty of Medicine, Asfendyarov Kazakh National Medical University, Almaty 050012, Kazakhstan; murtazina.a@kaznmu.kz (A.M.); tarabaeva.a@kaznmu.kz (A.T.); bitanova.e@kaznmu.kz (E.B.); 2 Biopathology and Regenerative Medicine Institute (IBIMER), Centre for Biomedical Research, University of Granada, 18016 Granada, Spain; yaijmartinez@correo.ugr.es (Y.J.-M.); glorual@correo.ugr.es (G.R.A.); jmarchal@ugr.es (J.A.M.); 3 Research Center “Bioscience Technologies”, Almaty 050057, Kazakhstan; 4 Instituto de Investigación Biosanitaria ibs. GRANADA, University Hospitals of Granada-University of Granada, 18014 Granada, Spain; 5 Department of Human Anatomy and Embryology, Faculty of Medicine, University of Granada, 18016 Granada, Spain; 6 Research Unit “Modeling Nature” (MNat), University of Granada, 18016 Granada, Spain; 7 Institute of Plant Biology and Biotechnology, Almaty 050040, Kazakhstan; ipbb_kz@yahoo.com; 8 Plant Biochemistry and Food Quality Group, Environmental and Biochemical Sciences Department, The James Hutton Institute, Invergowrie, Dundee DD2 5DA, UK; gordon.mcdougall@hutton.ac.uk; 9 Research Institute for Problems of Biology and Biotechnology, Al-Farabi Kazakh National University, Almaty 050040, Kazakhstan

**Keywords:** wheat cell culture, polysaccharide, colon cancer, cancer stem cells, proliferation

## Abstract

Natural polysaccharides (PSs) have shown inhibitory effects on differentiated cancer cells (DCCs), but their activity against cancer stem cells (CSCs) remains poorly understood. Here, we report that PSs from wheat cell cultures (WCCPSs) inhibit the proliferation of both DCCs and CSCs derived from HCT-116 colorectal cancer cells. Among them, NA and DC fractions showed the strongest anti-CSC activity. NA, rich in xylose, was effective at lower concentrations, while DC, enriched in xylose and galacturonic acid (GalUA), exhibited higher potency, with a lower IC_50_ and preferential activity against CSCs at higher doses. WCCPSs reduced β-catenin levels, and some fractions also downregulated Ep-CAM, CD44, and c-Myc. Notably, DC increased caspase-3 without inducing cytochrome C and caspase-8 overexpression, suggesting a mechanism promoting CSC differentiation rather than apoptosis. Correlation analysis linked xylose content to reduced c-Myc expression, and GalUA levels to increased caspase-3. These results suggest that WCCPS bioactivity may be related to their monosaccharide composition. Overall, our findings support the potential of wheat-derived PSs as CSC-targeting agents that suppress self-renewal and promote differentiation, offering a promising approach to reduce tumor aggressiveness and recurrence.

## 1. Introduction

Colon cancer ranks as the second leading cause of cancer-related deaths worldwide. In 2020, an estimated 1.9 million new cases of colorectal cancer (CRC) were diagnosed globally, along with over 930,000 deaths attributed to the disease. By 2040, the global burden of colorectal cancer is projected to rise significantly, reaching 3.2 million new cases (a 63% increase) and 1.6 million deaths (a 73% increase) annually [1]. Ineffective treatments of CRC are often caused by resistant cells with inherent stemness traits, not responding sufficiently to radiation therapy and chemotherapy, letting the disease spread to a metastatic phenotype [2,3]. In this regard, cancer stem cells (CSCs) have been shown to play a pivotal role in the development and resistance of CRC [4]. CSCs are a subpopulation of cells within tumors that possess self-renewal abilities, drive tumorigenesis, and contribute to metastasis, therapeutic resistance, and recurrence [5,6]. Due to their important role in cancer progression, CSCs represent a promising target for novel cancer therapies. In CRC, the persistence of CSCs is a significant challenge in treatment efficacy, as they are often resistant to conventional chemotherapies and can facilitate the spread of metastatic disease [7]. Therefore, identifying compounds capable of specifically targeting CSCs is of paramount importance in the development of more effective and targeted therapeutic strategies.

Finding out how stemness-related signaling pathways can control CSC behavior and whether they could be therapeutic targets to create more effective cancer fighting strategies have been investigated [8,9]. Identifying novel strategies for the efficient targeting of CSCs and elucidating their mechanisms of action through associated signaling pathways are crucial [8,9].

The search for novel compounds made from natural renewable resources for the safe and efficient treatment and prevention of cancer has gained more attention in recent years [10]. The use of plant, seaweed, mushroom extracts and agents derived from them – more frequently such metabolites as alkaloids, flavonoids, terpenoids, phenols, - has increased recently due to their capability to inhibit CSCs [10,11,12]. The studies that are currently available demonstrate how their anti-CSC characteristics mediate the modulation of multiple signaling pathways that are involved in the molecular and physiological regulation of CSCs [10,11,12,13,14,15]. It was shown that the stem-cell-like properties of CSCs are diminished by medicinal plants and bioactive compounds derived from plants, frequently by disrupting epithelial–mesenchymal transition (EMT) genes, decreasing invasiveness, and preventing CSCs from migrating [13]. Such plant-derived small molecules as Abrus agglutinin (lectin), Sanguinarine (alkaloid), and Ginsenoside-Rb1 (triterpenoid saponin) inhibit the Wnt/β-catenin signaling pathway; Baicalein (flavonoid), Curcumin (polyphenol), and Withaferin A (steroidal lactone) inhibit the Hedgehog signaling pathway; and Psoralidin (phenolic compound) inhibits the Notch signaling pathway in CSCs, suggesting their possible use in the reduction in resistance to chemotherapies [14].

The class of natural macromolecules known as PSs are thought to be promising candidates to fight cancer because of their non-toxic properties and antitumor properties [15]. PSs could be derived from plants, fungi, algae, and microorganisms [16,17]. Both in vitro and in vivo, they have demonstrated antitumor effects, immunomodulation, and antioxidant properties [15,16,17,18,19]. Much preclinical research has demonstrated that the biological activity of natural PSs can inhibit the growth of differentiated tumors and promote cell cycle arrest and apoptosis, increase the differentiation of cancer cells, decrease EMT, and modulate the immune response [20,21,22].

In the past ten years, some research has been conducted on the potential anticancer effect of natural PSs on CSCs. For instance, *Ganoderma lucidum* polysaccharide, a mushroom-derived PS, caused tumoral cells to behave in a less invasive and proliferative manner, suppressing EMT and CSC markers in oral squamous cell carcinoma [23]. Another PS extracted from *Trametes robiniophila Murr (Huaier)* mushroom exhibited anticancer effects by reducing mammosphere formation, lowering the expression of stem-cell-related genes, and decreasing the proportion of aldehyde dehydrogenase-positive cells in vitro. Additionally, it suppressed xenograft tumor formation in vivo in triple-negative breast cancer [24]. Seaweed PSs from *P. capillacea* and *C. officinalis* were found to downregulate *OCT4*, *SOX2*, *ALDH1A3*, and *vimentin* in MDA-MB-231; to suppress *Cyclin D1* gene expression in MDA-MB-231 and MCF-7 cells; and to downregulate *β-catenin* and *c-Myc* genes in MDA-MB-231 cells [25]. It was shown as well that carrageenan, a PS extracted from *Gigartina pistillata* seaweed, has antitumor potential against colorectal cancer stem-like cells [26]. PSs extracted from a plant *Lycium barbarum* reverse the drug resistance of colon CSCs when treated in combination with oxaliplatin, downregulating *Bcl2*, *ABCG2*, *PMI*, *PI3K*, and *AKT*, and therefore inhibiting the PMI/PI3K/AKT signaling pathway [27].

However, data on the effects of a wide variety of natural PSs on CSCs remain limited. The majority of investigations show the ability of natural PSs to suppress CSCs [23,24,25,26,27,28]; however, some research finds that natural PSs promote their growth and progression (for instance, chitosan—an amino polysaccharide) [29]. Therefore, elucidating the role of natural PSs in the biology of CSCs, considering the vast variety of dietary supplements containing PSs, is of special interest.

Our previous research demonstrated the anticancer effects of natural PSs derived from wheat cell culture (WCCPS) on the colorectal cancer HCT-116 cell line [22]. To advance our investigation, we expanded our analysis to include previously studied as well as additional samples of WCCPSs to evaluate their potential for inhibiting CSC proliferation. This study is particularly significant because the PSs were derived from wheat cell suspension cultures and cultivated wheat callus cells under standard laboratory conditions—an economically advantageous method for producing PSs using the widely available *Triticum aestivum* variety. Based on these findings, we propose that WCCPSs could serve as a promising and cost-effective natural preventive or adjunct therapeutic agent for colon cancer treatment.

## 2. Materials and Methods

### 2.1. Plant Cell Culture, Purification, and Separation of PSs

Cell suspension cultures of the soft spring wheat *Triticum aestivum*, variety Kazakh-stanskaya 10 (supplied by the Kazakh Research Institute of Agriculture and Plant Growing, located in Almaty, Kazakhstan), were cultivated in three distinct types of Murashige and Skoog (MS) [30] liquid medium (Sigma-Aldrich, St. Louis, MO, USA), as previously described [31]. The cultures were supplemented with phytohormones, which are plant growth regulators, commonly used in plant biotechnology to modulate the morphogenesis and production of secondary bioactive metabolites in vitro [32,33,34,35]. Specifically, the medium contained 5.0 mg/L of 2,4-D (2,4-dichlorophenoxyacetic acid) and 0.5 to 1.0 mg/L of ABA (abscisic acid), both from Sigma-Aldrich, St. Louis, MO, USA.

To generate cell suspension cultures, 200 mg of wheat callus tissue, initially grown on solid medium, was transferred to 30 mL of liquid culture medium in laboratory flasks (Sigma-Aldrich, St. Louis, MO, USA) and incubated in an ES-20 Incubator Shaker (BioSan, Riga, Latvia) at 140 rpm and 26 ± 2 °C under a 16 h photoperiod with a light intensity of 10 μmol m^−2^ s^−1^ for periods of one, two, and four weeks. The resulting cell culture media were then filtered using Whatman qualitative filter paper (Merck, Darmstadt, Germany), concentrated using a rotor evaporator (IKA, RV 3 V, Staufen, Germany), and extracellular PSs were precipitated with ethanol. For the obtainment of cellular PSs, filtered cells from suspension were dried (1 g) under laminar flow and then extracted with water (1 L) at 50 °C for two hours until PSs were no longer detected [36,37,38,39]. Generally, three replicate extractions were obtained, combined, and then concentrated by rotor evaporation to a minimal volume.

Preparation and Purification of PSs: Extracellular and cellular PSs were precipitated from the media and water extracts, respectively, by adding 70% (*v*/*v*) ethanol (Sigma-Aldrich, St. Louis, MO, USA) and incubating the mixture at 4 °C for a minimum of 4 h. The resulting PS pellets were collected through centrifugation at 10,000 rpm for 10 min at 8 °C using a refrigerated centrifuge (5810R, Eppendorf, Hamburg, Germany). To confirm the enrichment of total sugar content, the Dubois method [39] was employed, with measurements taken using a SmartSpec spectrophotometer (Biorad, Hercules, CA, USA). The precipitated PSs were then resuspended in ultrapure water (<18 MΩ, Elga Water Systems, High Wycombe, UK) and freeze-dried using a Martin Christ ALPHA 1–2 LD plus freeze dryer (Osterode am Harz, Germany).

### 2.2. Characterization of PS Samples and Analysis of Monosaccharide Content

**Figure 1:**
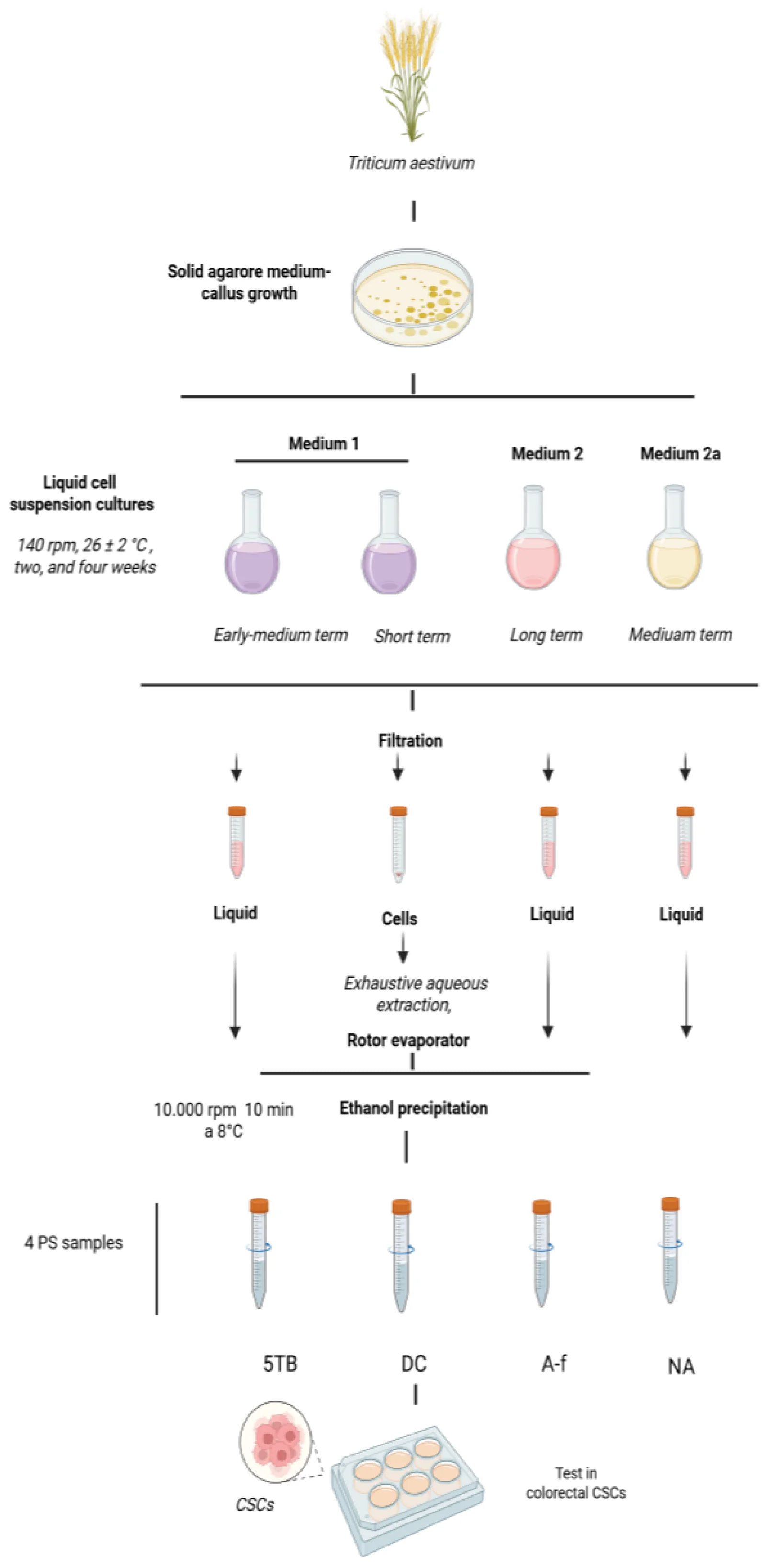
Scheme for the obtainment of WCCPS samples.

A total of five distinct PS fractions were obtained, each varying based on the nutrient media, cellular or extracellular origin, and cultivation duration (Figure 1). Wheat callus cells were cultured in two different liquid media: medium 1 supplemented with 5.0 mg/L of 2,4-D; medium 2 containing 1.0 mg/L of ABA; and medium 2a with 0.5 mg/L of ABA [31]. The WCCPS samples utilized in this study were derived from various cultivation periods: short-term (7 days), early-medium-term (14 days), medium-term (21 days), and long-term (42 days). The short-term culture yielded the DC sample, the early-medium-term culture produced the 5TB sample, the medium-term culture provided the NA sample, and the long-term culture generated the A-f fraction. Additionally, 10 other samples were tested on CSCs but did not exhibit significant effects and, therefore, were excluded from this manuscript.

The monosaccharide composition of the PS fractions was analyzed using acid hydrolysis, followed by high-performance anion exchange chromatography (HPAEC) [40]. In brief, 10 mg of each PS fraction was hydrolyzed in triplicate with 1 mL of 2 M trifluoroacetic acid (Sigma-Aldrich, St. Louis, MO, USA) at 120 °C for 2 h. After centrifugation (10,500× *g*, 5 min), the supernatant was evaporated in a Speed-Vac (Genevac Ltd. miVac Duo, Ipswich, UK) and the residue was resuspended in ultrapure water. After appropriate dilution, the monosaccharide composition was determined by comparing it with standard curves of relevant monosaccharides, which were separated using a CarboPAC PA20 column and detected via pulsed amperometric detection (Dionex ICS-5000 system, Thermo Fisher Scientific Inc., Waltham, MA, USA). All polysaccharide fractions were initially dissolved in ultrapure water with 0.05% DMSO to prepare stock solutions. For experimental purposes, stock solutions were diluted in DMEM to achieve the required concentrations.

### 2.3. Cell Lines

The HCT-116 colon cancer cell line was sourced from the American Type Culture Collection (ATCC, Manassas, VA, USA) and cultured in Dulbecco’s Modified Eagle’s Medium (DMEM; Sigma-Aldrich, St. Louis, MO, USA), supplemented with 10% fetal bovine serum (FBS) and 1% penicillin/streptomycin. All cell lines were authenticated through short-tandem repeat (STR) profiling and were passaged for a maximum of 6 months. Regular mycoplasma contamination checks were also performed on the cultures.

### 2.4. Obtainment of CSCs

#### 2.4.1. Sphere-Forming Assay

To assess the self-renewal capacity of the HCT-116 cell line population, a sphere-forming assay was conducted to isolate enriched HCT-116 CSC subpopulations. These subpopulations were obtained by culturing primary and secondary spheroids in serum-free medium under anchorage-independent conditions, as follows: 2.5 × 10^5^ adherent cells were collected, washed with PBS, and resuspended in sphere culture medium (DMEM:F12, 1% penicillin/streptomycin, B27, 10 μg/mL of ITS, 1 μg/mL of hydrocortisone, 4 ng/mL of heparin, 10 ng/mL of EGF, 20 ng/mL of FGF) in ultra-low-adherence 6-well plates (Corning). After 3 days, the primary spheres were harvested, washed with PBS, and dissociated using a sterile 25-gauge needle. Subsequently, 2.5 × 10^5^ cells were replated and resuspended in sphere culture medium in ultra-low-adherence 6-well plates (wo 2016/020572 A1) [41].

#### 2.4.2. Colony-Formation Assay

The clonogenic potential of HCT-116 CSCs was evaluated using a colony formation assay in soft agar, following established protocols [41,42]. In brief, 10^4^ cells derived from secondary spheroids were plated onto a 0.4% cell agar suspension layer, which was on top of a 0.8% base agar layer in 6-well culture plates. The cultures were incubated at 37 °C in a humidified atmosphere with 5% CO_2_ for 21 days. After the incubation period, cell colonies were stained with 0.1% iodonitrotetrazolium chloride (Sigma-Aldrich) and visualized under a light microscope.

#### 2.4.3. ALDEFLOUR Assay and Phenotypic Characterization by Flow Cytometry

The expression of CD326 (APC) and CD44 (PE) surface markers, as well as ALDH1 activity, was evaluated using a Becton Dickinson FACSCanto II Flow Cytometer at the CIC Scientific Instrumental Centre (University of Granada). ALDEFLUOR assays (Stem Cell Technologies, Vancouver, BC, Canada) were utilized to measure ALDH1 activity in viable cells, alongside the assessment of cell surface expression levels of CD326 and CD44 (Miltenyi Biotec, Bergisch Gladbach, Germany). These assays were conducted following the protocols provided by the manufacturer.

#### 2.4.4. Side Population Analysis

The side population (SP) was defined as the subset of cells capable of effluxing Hoechst 33342, as indicated by their distinct fluorescence pattern. Hoechst 33342 exclusion assays were carried out as previously described [42] to assess cells that overexpress ABC transporters. Single-cell suspensions from both parental cell lines and HCT-116 CSCs were stained with Hoechst 33342 dye (Sigma-Aldrich). Verapamil (Sigma-Aldrich) was utilized as a negative control to block efflux activity, thereby preventing cells from expelling Hoechst 33342. Flow cytometric analysis was performed using a FACScan Aria III system (BD Biosciences, San Jose, Palo Alto, California, 95131), measuring Hoechst blue (440/40) and Hoechst red (695/40) emission spectra. Data collection and analysis were performed using FACSDIVA software (version 7.1) at the CIC Scientific Instrumental Centre (University of Granada).

### 2.5. In Vitro Cytotoxicity Analysis

The impact of PS fractions on cell viability was measured using the Thiazolyl Blue Tetrazolium Bromide (TBTB) colorimetric assay (MTT). Briefly, 2.5 × 10^3^ cells per well were plated in 96-well plates, allowed to incubate for 24 h, and then exposed to various concentrations of PS fractions. After 72 h, the medium was removed, and the cells were incubated with TBTB reagent for 3 h, followed by the addition of dimethyl sulfoxide (DMSO) (≥99.5%) to dissolve the formazan product. Cell viability was then quantified using a Titertek Multiscan apparatus (Flow, Irvine, CA, USA) at 570 nm, as described previously [43].

### 2.6. Western Blotting

The CSCs obtained from the HCT-116 cell line were seeded in 6-well plates in DMEM. After 48 h of treatment with an X2 IC_50_ concentration for each sample, the culture medium was removed, and the cells were collected by centrifugation at 1500 rpm.

Afterward, the cells were rinsed twice with PBS and subjected to lysis using RIPA lysis buffer (Santa Cruz Biotechnology, Dallas, TX, USA). Immunoblotting of the whole cell lysates was carried out following conventional protocols [44]. The primary antibodies employed for protein detection included caspase-3, diluted 1:1000 (Cell Signaling, Beverly, MA, USA, #9662); caspase-8, diluted 1:500 (Santa Cruz Biotechnology, Dallas, TX, USA, sc-56070); cytochrome-c, diluted 1:500 (Santa Cruz Biotechnology, Dallas, TX, USA, sc-13560); β-actin, diluted 1:15,000 (Sigma-Aldrich, St. Louis, MO, USA, A2228); c-Myc, diluted 1:100 (Santa Cruz Biotechnology, Dallas, TX, USA, sc-40); beta-catenin, diluted 1:100 (Santa Cruz Biotechnology, Dallas, TX, USA, sc-57533); CD44, diluted 1:200 (Santa Cruz Biotechnology, Dallas, TX, USA, sc-7297); and EpCAM, diluted 1:100 (Abcam, Cambridge, UK, ab85987). For secondary detection, anti-rabbit IgG peroxidase conjugate (Sigma-Aldrich, St. Louis, MO, USA, A0545) and anti-mouse IgG peroxidase conjugate (Sigma-Aldrich, St. Louis, MO, USA, A9044) were used. The protein–antibody complexes were visualized using enhanced chemiluminescence (ECL, Bonus, Amersham, Little Chalfont, UK) and detected through the ImageQuant LAS 4000 mini (GE Healthcare, Chicago, IL, USA). Signal intensity was quantified using Image J software (version 1.53a). The band intensities were normalized by dividing them by the corresponding β-actin value and relativized to the control sample, which was assigned a value of 1. Two separate membranes were used for protein detection: Beta-catenin (92 kDa), c-Myc (67 kDa), and caspase-3 (35 kDa) were analyzed on one membrane, while the second membrane was used for CD44 (90–95 kDa), caspase-8 (55 kDa), EpCAM (33–40 kDa), and Cyt C (14 kDa). Each experiment was performed at least three times.

### 2.7. Statistical Analysis

All experiments were performed in at least three replicates. The results are presented as means ± standard deviation (SD). Data normality was assessed using the Shapiro–Wilk and Kolmogorov–Smirnov tests. To determine the statistical significance of differences between the two groups, Student’s *t*-test was applied. A *p*-value of 0.05 or less was considered statistically significant. Figures were generated using GraphPad Prism 8 software. The 50% inhibition concentration (IC_50_) values were calculated using various methods due to non-monotonic concentration–response relationships or failure to achieve IC_50_. To estimate the predicted IC_50_, we employed a four-parametric logistic dose–response model with a non-linear logarithmic fit and variable slope. To evaluate the correlation between monosaccharide content/proportion in WCCPS samples and protein expression level in CSCs, we used the Pearson correlation coefficient, following the approach of Han et al. [45].

## 3. Results

### 3.1. Monosaccharide Composition of WCCPSs

The relative monosaccharide composition of WCCPS samples determined by HPAEC is depicted in Table 1. Glucose suggests the presence of glucans; arabinogalactans suggest the presence of arabinose and galactose; glucurono- and galacturonoarabinoxylans suggest the presence of glucuronic and galacturonic acids and xylose; and xylose and glucose also indicate the presence of xyloglucans [22].

The monosaccharide (MS) composition of the NA sample was composed mainly of xylose (52%) and glucose (34.6) and had the following ratio—Ara: Xyl: Gal: Glc: GlcUA: GalUA: Man = 5: 52: 3: 35: 1: 2: 1 (Table 1).

The content of MS in the A-f sample was glucose (83%), arabinose (7%), galactose (3%), and xylose (5%) and had the following ratio—7: 5: 3: 83: 1: 0.1: 0 [22].

The DC sample consisted of xylose (40%), glucose (33%), and galacturonic acid (21%) with the following MS ratio, 2: 40: 2: 33: 1: 21: 1.

Sample 5TB was composed of arabinose (19.2), xylose (15.9%), galactose (8.3%), glucose (15.9%), and glucuronic acid (6%) and had the following MS ratio—Ara: Xyl: Gal: Glc: GlcUA: GalUA: Man = 20: 16: 8: 16: 3.5: 0: 0.

Overall, the NA sample was primarily composed of xylose (52%) and glucose (35%), suggesting enrichment in xyloglucans but with some arabinose, galactose, and galacturonic acid. The A-f sample had a high level of glucose (83.2%), which suggests the presence of beta-glucans along with 7% arabinose and 4.8% xylose. The DC sample also exhibited characteristics of xyloglucan (40% xylose and 33% glucose) with a significant presence of galacturonic acid (21%) and low arabinose, classifying it as a galacturonan. The monosaccharide profile of the 5TB sample suggests a mixture of arabinoxylans, arabinogalactans, and xyloglucans with some glucuronic acid.

### 3.2. Characterization of CSC-Derived Colonospheres

To enrich CSCs, the established HCT-116 colon cancer cell line was employed. The anchorage-independent growth of HCT-116 cells under serum-free conditions was evaluated to confirm their CSC characteristics. This was achieved by assessing their clonogenic capacity through soft agar colony formation assays, sphere formation ability, side population analysis, ALDH1+ activity, and the expression of CD326 and CD44 cell surface markers. HCT-116 cells cultured under anchorage-independent and serum-free conditions formed spheres with a consistent, rounded morphology, albeit in lower numbers compared to adherent cells. This observation is consistent with the slow-cycling nature typically associated with stem cell populations [46,47].

**Figure 2:**
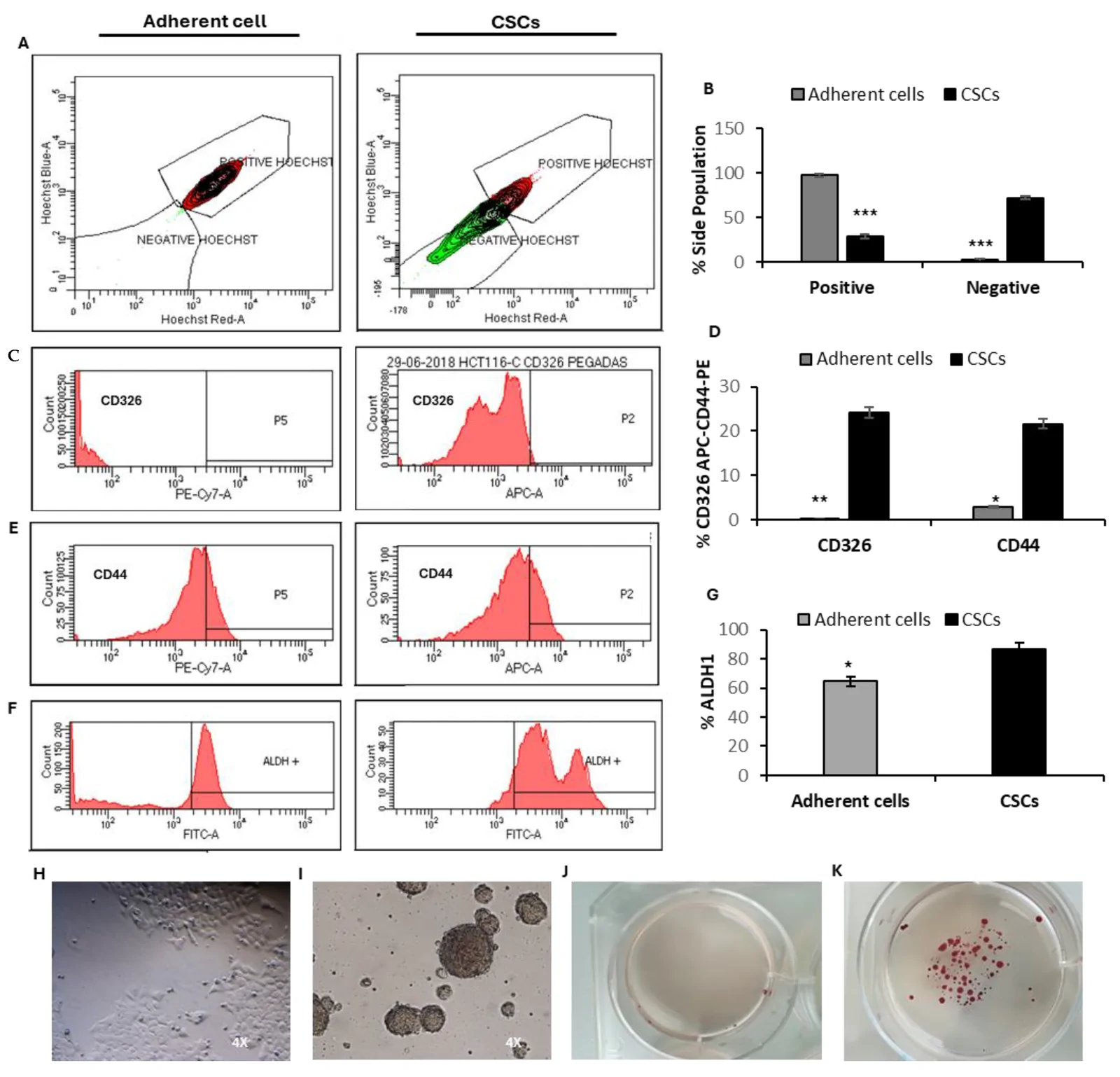
Characterization of HCT-116 CSCs. (**A**,**B**) Side population analysis in HCT-116 adherent cells and secondary CSCs. (**C**–**E**) The percentage of CD326 and CD44 expression in adherent cells and secondary CSCs from the HCT-116 cell line. (**F**,**G**) The proportion of ALDH1+ cells assessed by flow cytometry. (**H**,**I**) Representative light microscopy images (4×) of adherent cells (left) and secondary CSCs (right) derived from the HCT-116 cell line. (**J**,**K**) Optical images showing colonies formed by HCT-116 cells originating from adherent cells and secondary spheroids after 21 days of soft agar culture in P6 well plates, stained with 0.1% iodonitrotetrazolium chloride. Data are presented as means ± SD from triplicate experiments (*** *p* < 0.001; ** *p* < 0.01; * *p* < 0.05).

Soft agar colony formation assays are a standard method for assessing the ability of cells to proliferate in an anchorage-independent manner, which is a key feature of CSCs. In this assay, cells capable of forming colonies in a semi-solid medium demonstrate clonogenic potential and a degree of self-renewal associated with stem-cell-like behavior [48,49]. This technique compared HCT-116 cells cultured as colonospheres with their adherent counterparts. Colonospheres exhibited a significantly enhanced colony-forming capacity compared to adherent cells, highlighting their increased clonogenic potential and CSC-like properties (Figure 2J,K).

Consistently, the proportion of side-population-negative cells in secondary spheres was significantly higher than in adherent cells, with 72.2% observed in secondary spheres compared to only 2% in adherent cells (Figure 2A,B). Secondary spheres also displayed a marked increase in the expression of the surface markers CD326 and CD44, with 24.2% and 21.6% of cells expressing these markers, respectively, compared to just 0.3% and 2.9% in adherent cells (Figure 2C–E). Furthermore, in terms of ALDH1+ activity, HCT-116 spheres exhibited a significantly higher proportion of ALDH+ cells (86.4%) compared to adherent cells (64.5%) (Figure 2F,G).

Taken together, these results indicate that HCT-116 cells cultured as secondary spheres under anchorage-independent and serum-free conditions are enriched for functional and phenotypic CSC properties. Given that secondary spheres demonstrated the highest enrichment of CSC characteristics, they were selected for subsequent studies.

### 3.3. Inhibition of Proliferation of HCT-116 Differentiated Cancer Cells

**Figure 3:**
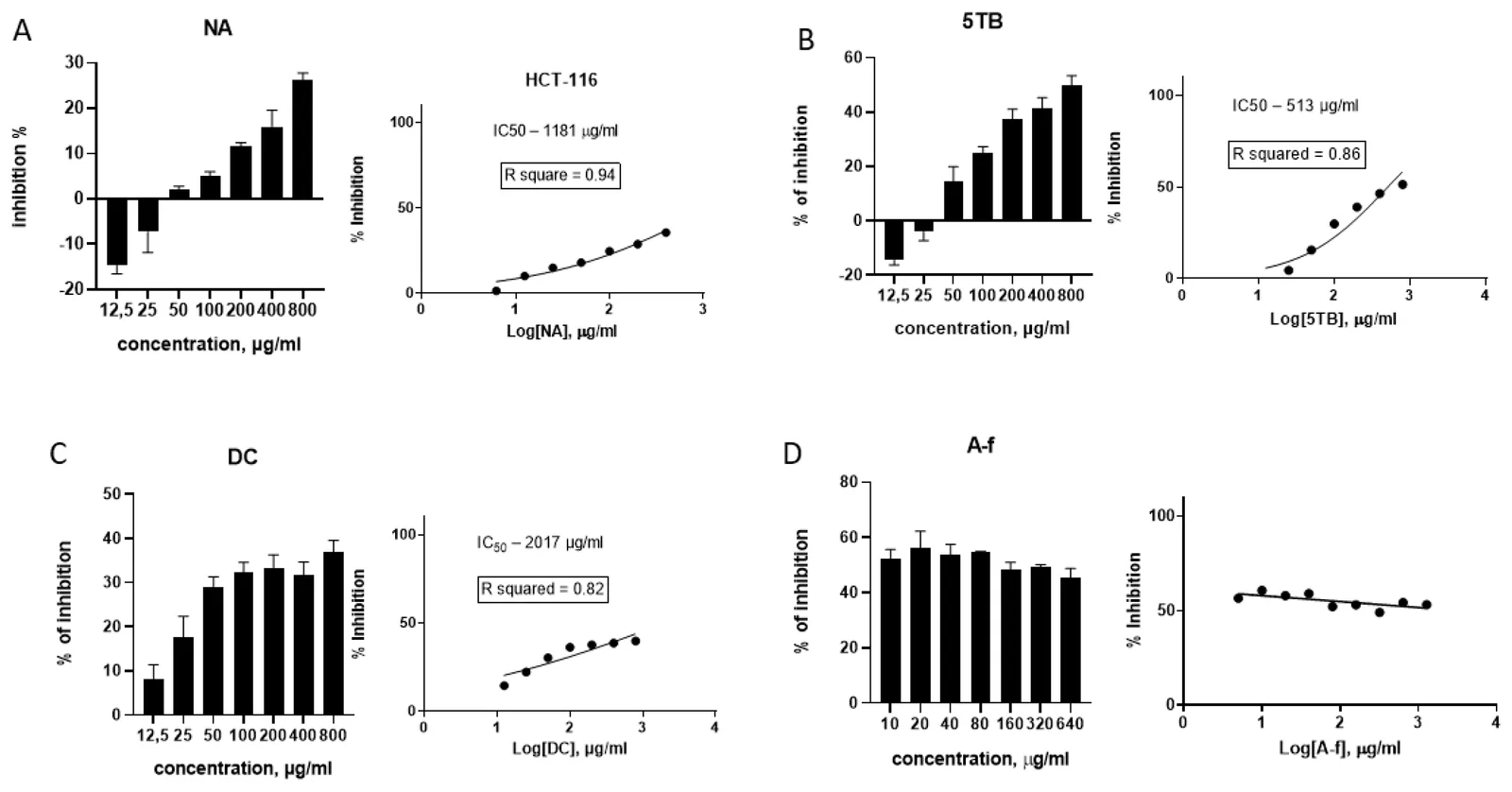
Effects of WCCPSs on the proliferation of HCT-116 colon cancer cells. (**A**) NA sample; (**B**) 5TB sample; (**C**) DC sample; and (**D**) A-f sample (published result [22]). Data are graphed as the mean ± SD from experiments carried out in triplicates.

WCCPS fractions effectively inhibited the growth of HCT-116-colon-differentiated cancer cells (DCCs) across different concentrations. The NA sample inhibited cancer cell proliferation from 50 µg/mL to 800 µg/mL, with inhibition rates varying from 2% ± 0.6 to 23.7% ± 1.5, respectively. The inhibitory concentration for 20% of cells (IC_20_) for the NA sample was approximately between 400 and 800 µg/mL, trending closer to 800 µg/mL. To estimate the concentration at which 50% of cells were inhibited (IC_50_), logarithmic dose–response curves with non-linear interpolation were used and the IC_50_ for NA on the HCT-116 cell line was 1181 µg/mL (R square = 0.94 estimate) (Figure 3A).

The 5TB sample exhibited inhibitory effects on cancer cells starting at 50 µg/mL (14.4% ± 5.4) and reaching 50.4% ± 3.53 at 800 µg/mL. The use of logarithmic dose–response analysis allowed exact values of IC_50_ to be determined, which was estimated to be 513 µg/mL (Figure 3B).

The DC sample inhibited the growth of HCT-116 cells at 12.5 µg/mL (8.2% ± 3.1), reaching approximately 30% inhibition (IC_30_) at concentrations between 50 and 100 µg/mL. The inhibition rate remained at around 30% from 100 to 400 µg/mL, increasing to 36.9% ± 2.7 at 800 µg/mL. Based on the logarithmic dose–response curve, 50% inhibition was estimated at 1582 µg/mL (Figure 3C).

The A-f fraction started demonstrating an IC_50_ level of 10 µg/mL, as described previously [22]. Since the extent of inhibition remained stable at approximately 50% regardless of the concentration increase from 10 to 640 µg/mL, non-linear extrapolation was not applicable for this sample (Figure 3D).

### 3.4. Inhibition of Proliferation of CSCs

**Figure 4:**
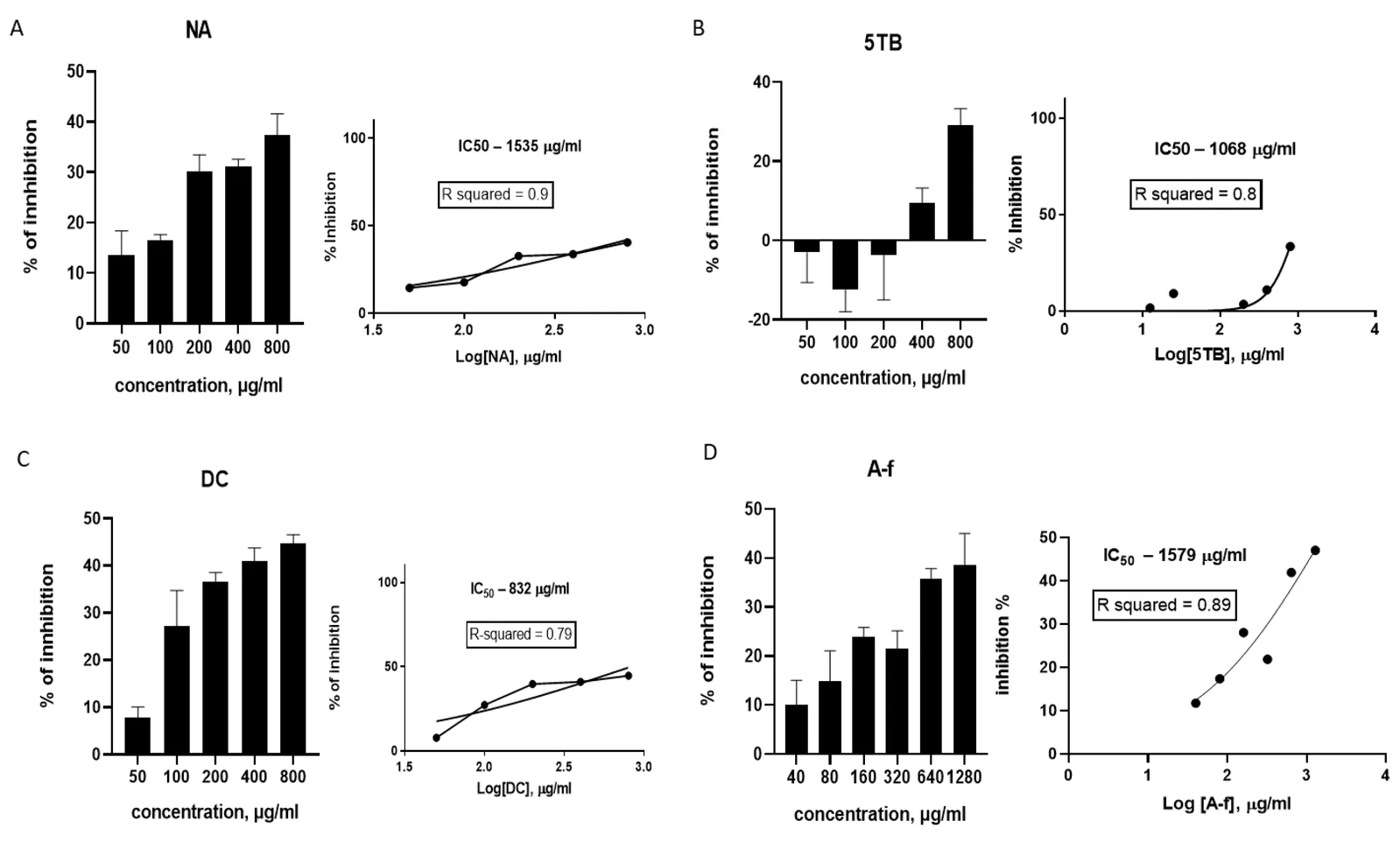
Effects of WCCPSs on the proliferation of CSC from the HCT-116 cell line: (**A**) NA sample; (**B**) 5TB sample; (**C**) DC sample; and (**D**) A-f sample.

The samples inhibited CSC proliferation at various concentrations (Figure 3). The NA sample exhibited inhibitory activity across all tested concentrations. Specifically, within the concentration range of 50 to 100 µg/mL, inhibition was at the same level—13% ± 4.8 to 16.4% ± 1.2. Between 200 and 800 µg/mL, NA inhibited cell proliferation by 30.1% ± 3.2 to 37.3% ± 4, respectively. Consequently, the IC_35_ for this sample was determined to be 800 µg/mL. The IC_50_ value was estimated at 1535 µg/mL based on the logarithmic dose–response curve (Figure 4A).

The 5TB sample stimulated CSC proliferation between 50 and 200 µg/mL. However, 400 and 800 µg/mL of 5TB inhibited cell proliferation by 9.4% ± 3.8 and 29% ± 4.2, respectively. Consequently, the approximate IC_30_ for this sample was 800 µg/mL. The logarithmic dose–response curve estimated the IC_50_ at 1068 µg/mL (Figure 4B).

The DC sample exhibited the highest inhibitory activity against CSCs. At 50 µg/mL, 9% ± 5 of the cells were inhibited. At 100–200 µg/mL, inhibition increased from 27% ± 7 to 36.6% ± 1.9, with the further increase in inhibition up to 40.9–44.6% consequently observed at higher concentrations of 400–800 µg/mL. Therefore, the IC_35_ for this sample was 200 µg/mL. The dose–response curve estimated for DC sample by linear extrapolation indicated an IC_50_ of 832 µg/mL (Figure 4C).

All concentrations of the A-f sample exhibited an inhibitory effect on CSCs, starting from 40 µg/mL (10% ± 5 inhibition) up to 1280 µg/mL (38.6% ± 6.4 inhibition), with an IC_35_ value of 640 µg/mL (Figure 4D). The IC_50_ for the A-f sample was estimated to be 1579 µg/mL by linear extrapolation.

### 3.5. Comparison of WCCPS Samples on DCCs and CSCs of HCT-116 Cell Line

**Figure 5:**
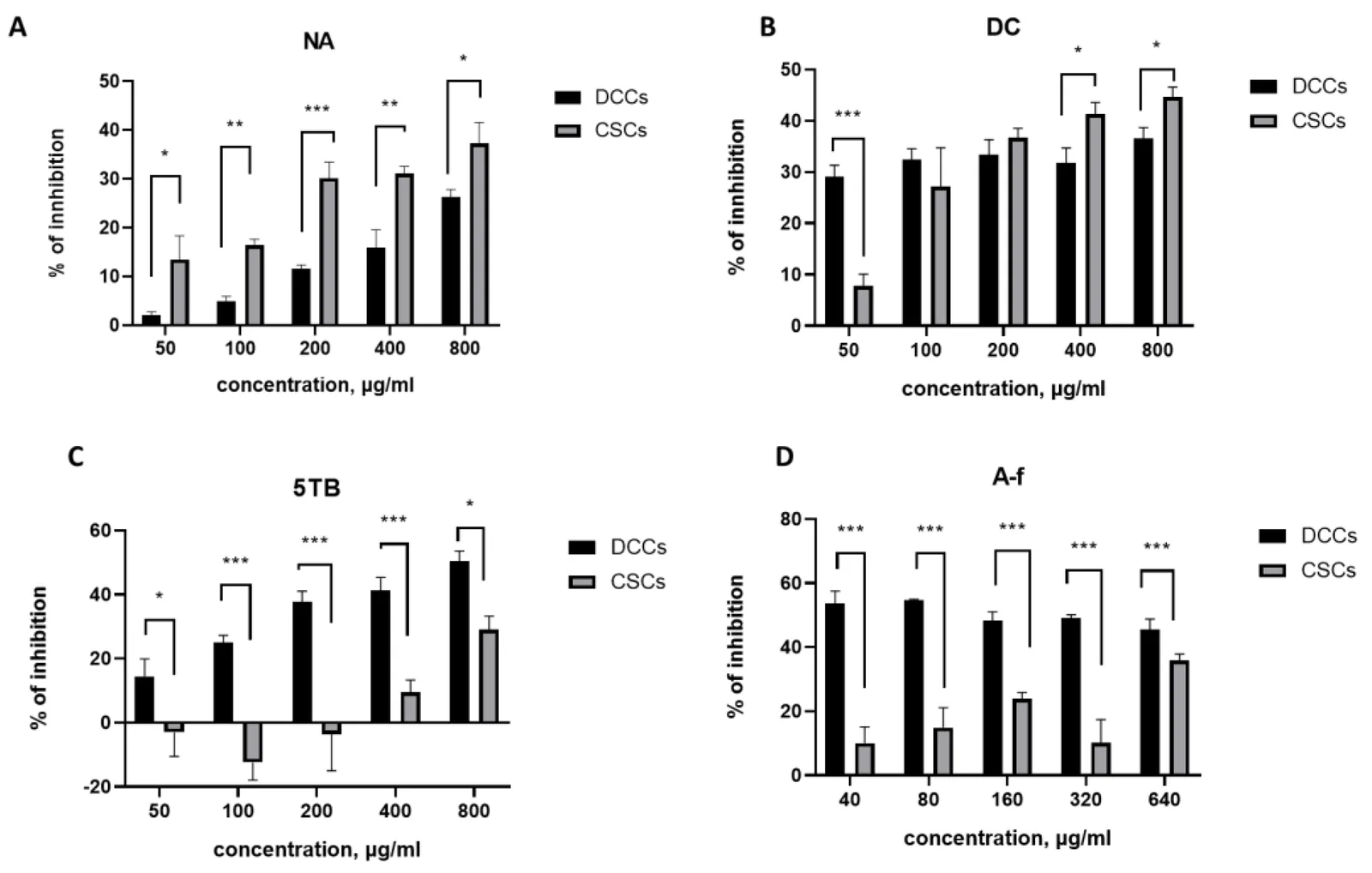
Inhibition of CSC and DCC line proliferation by WCCPS samples. (**A**). NA sample, (**B**). DC sample, (**C**). 5TB sample, and (**D**) A-f sample (*** *p* < 0.001, ** *p* < 0.01, and * *p* < 0.05).

Analysis of the data revealed that two samples, NA and DC, were more effective at inhibiting HCT-116 CSCs than DCCs, whereas the 5TB and A-f samples displayed greater efficacy against DCCs (Figure 5).

For example, the NA sample inhibited 25–30% of CSCs at a concentration four times lower (IC_30_ = 200 μg/mL) than that required to achieve a comparable inhibition of DCCs (IC_25_ = 800 μg/mL). Moreover, across all tested concentrations (50–800 μg/mL), the NA sample induced a significantly higher level of inhibition in CSCs (13.5–37.3%) compared to DCCs (2.1–26.3%). These results indicate that NA exerts a stronger inhibitory effect on CSCs than on DCCs. Notably, the inhibition of both cell types increased proportionally with the NA concentration (Figure 5A).

In contrast, the DC sample exhibited a distinct dose-dependent response (Figure 5B). At the lowest tested dose (50 μg/mL), DC significantly inhibited DCCs (29%) compared to CSCs (7.7%). At intermediate concentrations (100 and 200 μg/mL), both CSCs and DCCs were inhibited to a similar extent, 32.3% and 33.3% for DCCs, and 27.1% and 36.6% for CSCs, respectively, with no statistically significant differences. At higher concentrations (400 and 800 μg/mL), the DC sample preferentially inhibited CSCs over DCCs. Specifically, at 400 μg/mL, inhibition reached 40.9% for CSCs and 31.1% for DCCs, while at 800 μg/mL, CSC inhibition rose to 44.6% compared to 37.3% for DCCs. These results suggest that DC exerts a stronger inhibitory effect on DCCs at low concentrations (50 μg/mL), a comparable effect on both cell types at intermediate concentrations (100–200 μg/mL), and a more pronounced effect on CSCs at higher concentrations (400–800 μg/mL).

The other two samples, A-f and 5TB, were more effective against DCCs than colonospheres (Figure 5C,D). The A-f sample exhibited an IC_50_ of 10 μg/mL for DCCs, while the corresponding value for CSCs was significantly higher (1579 μg/mL). Similarly, the 5TB sample showed an IC_50_ of 513 μg/mL for DCCs and 1068 μg/mL for CSCs.

**Figure 6:**
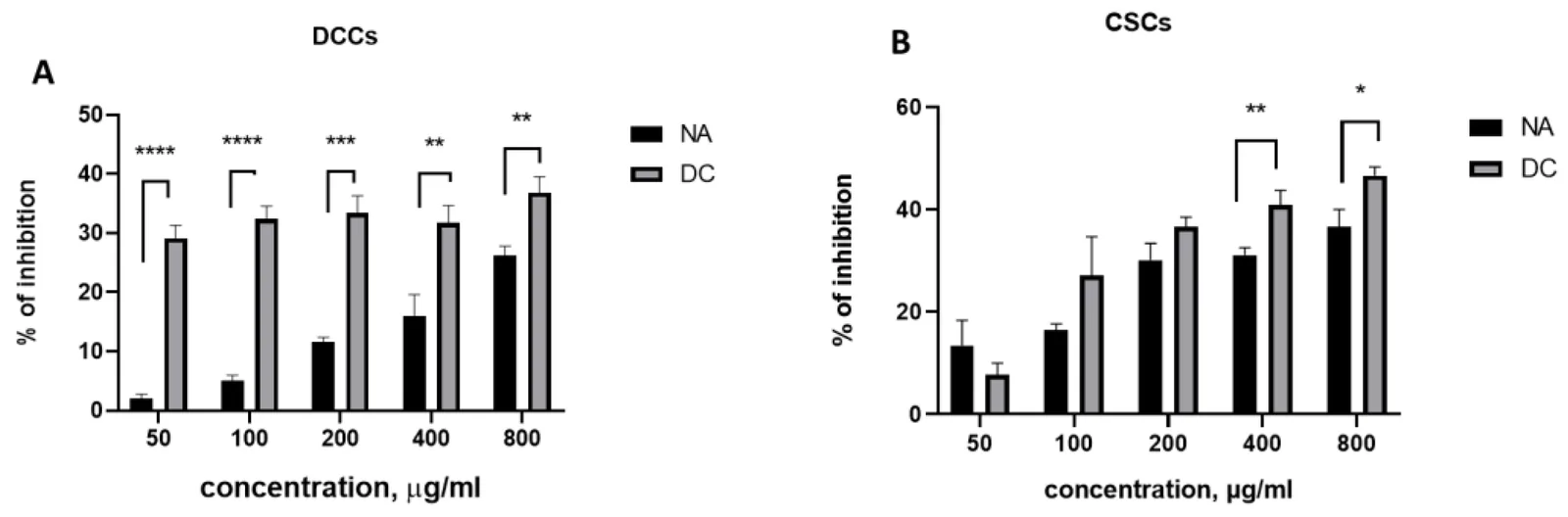
Comparison of inhibitory effect of NA and DC samples on DCCs (**A**) and CSCs (**B**). (**** *p* < 0.0001, *** *p* < 0.001, ** *p* < 0.01, and * *p* < 0.05).

A comparative analysis of the inhibitory effects of NA and DC samples’ DCCs and CSCs (Figure 6) revealed distinct inhibition profiles. Although both samples exhibited inhibitory activity against CSCs, their patterns of action differed. The NA sample consistently reduced DCC viability across all tested concentrations (Figure 6A), whereas the DC sample demonstrated a more pronounced inhibition of CSCs at higher concentrations (Figure 6B). In the intermediate concentration range (50–200 μg/mL), both samples induced comparable levels of CSC inhibition (Figure 6B).

Overall, the DC sample inhibited DCC proliferation by 30–35% across the entire concentration range tested (50–800 μg/mL), while CSC inhibition was only significant at concentrations between 100 and 800 μg/mL, with inhibition rates ranging from 25% to 45%. The maximum inhibitory effects observed for both cell types were greater with the DC sample (35–45%) compared to the NA sample (25–35%). These results highlight the DC fraction as a promising candidate for therapeutic applications, particularly at higher concentrations (400 and 800 μg/mL), where it effectively suppresses both CSCs and DCCs.

### 3.6. Mechanism of Anticancerous Action

**Figure 7:**
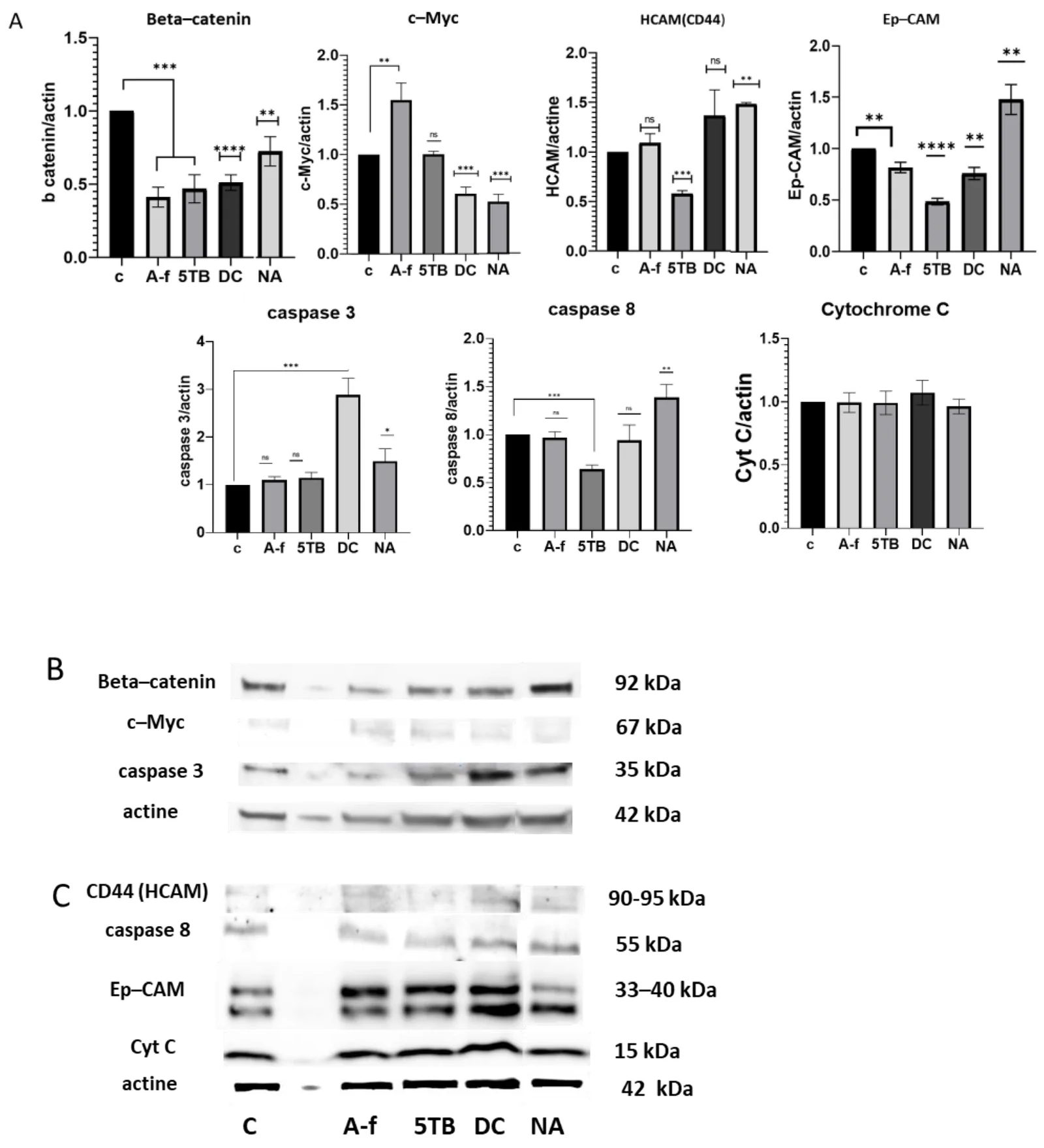
The impact of WCCPSs on protein expression in CSCs derived from the HCT-116 cell line. Western blot quantification was normalized to the β-actin signal. The data were collected from three independent experiments conducted in duplicate and are presented as means ± SD from three independent experiments performed in triplicate (**** *p* < 0.0001, *** *p* < 0.001, ** *p* < 0.01, and * *p* < 0.05 compared to control). (**A**) A graphical representation of protein levels (A-f, 5TB, DC, NA). (**B**) The first membrane (beta-catenin, c-Myc, caspase-3). (**C**) The second membrane (CD44, caspase-8, EpCAM, Cyt C). ns：not significant.

Levels of proteins that are known to be involved in cancer cell stemness development were evaluated by Western blotting after the treatment of CSCs with WCCPS fractions (Figure 7).

- Beta-catenin: All samples showed the capacity to reduce the levels of beta-catenin expression. Compared to the control value (1), A-f decreased levels to 0.4-fold, 5TB to 0.46-fold, DC to 0.5-fold, and the NA sample to 0.72-fold (Figure 7). This decrease in beta-catenin suggests that all samples contribute to the depletion of EMT processes in CSCs.
- c-Myc: C-Myc levels were compared to the control value (1). The treatment of CSCs with A-f resulted in an increase in c-Myc levels to 1.55-fold. 5TB did not show any effect on this protein. In contrast, DC and NA samples significantly reduced c-Myc levels to 0.6- and 0.51-fold, respectively (Figure 7). These three samples may be involved in reducing cell proliferation and promoting differentiation in CSCs.
- HCAM (CD44): The 5TB sample reduced CD44 protein levels, with a relative value of 0.42-fold, suggesting its potential to promote CSC differentiation. Conversely, the NA sample increased HCAM (CD44) levels, reaching a value of 1.51-fold (Figure 7). No significant effect on CD44 protein levels was observed with the DC and A-f samples in CSCs.
- Ep-CAM: After treatment with WCCPSs, Ep-CAM levels were decreased in four of the samples. A-f reduced the relative value to 0.8, 5TB to 0.48, and DC to 0.76 compared to the control (1). In contrast, the NA sample increased Ep-CAM levels to 1.47-fold (Figure 7). Therefore, the majority of samples inhibited Ep-CAM protein expression, suggesting their potential to reduce stemness, EMT, angiogenesis, and metastasis, as Ep-CAM plays a crucial role in initiating these processes.
- Cytochrome C: The levels of cytochrome C remained unchanged across all WCCPS samples tested (Figure 7), suggesting that none of these treatments triggered its release in CSCs.
- Pro-caspase-3: The DC sample caused a significant increase in pro-caspase 3 levels, nearly tripling the value compared to the control (relative value: 2.9 vs. 1). In contrast, the NA sample produced a more moderate increase (relative value: 1.5), while the other samples did not induce notable changes (Figure 7).
- Caspase-8: 5TB reduced caspase-8 levels, with relative values of 0.48. In contrast, NA slightly increased caspase-8 levels to 1.48-fold compared to the control (1) (Figure 7). No significant effects on caspase-8 expressions were observed for the DC and A-f samples.

It was determined that WCCPSs have the ability to take part in important cellular signaling processes in CSC development, altering key proteins of EMT, stemness, and differentiation. Thus, all investigated WCCPS samples described reduced beta-catenin levels. Three samples (DC, NA) decreased c-Myc levels. 5TB decreased levels of CD44 protein. Almost all samples decreased levels of Ep-CAM protein. These findings suggest that even though all samples have the capability to downregulate the EMT pathway, each sample had its own unique regulatory effect, influencing some proteins differently. With regard to the ability of WCCPSs to induce apoptosis of CSCs, there was only one sample, DC, that dramatically increased pro-caspase-3 levels, while not increasing cytochrome C release. 

### 3.7. Relationships Between Monosaccharide Composition and Protein Expression Levels

**Figure 8:**
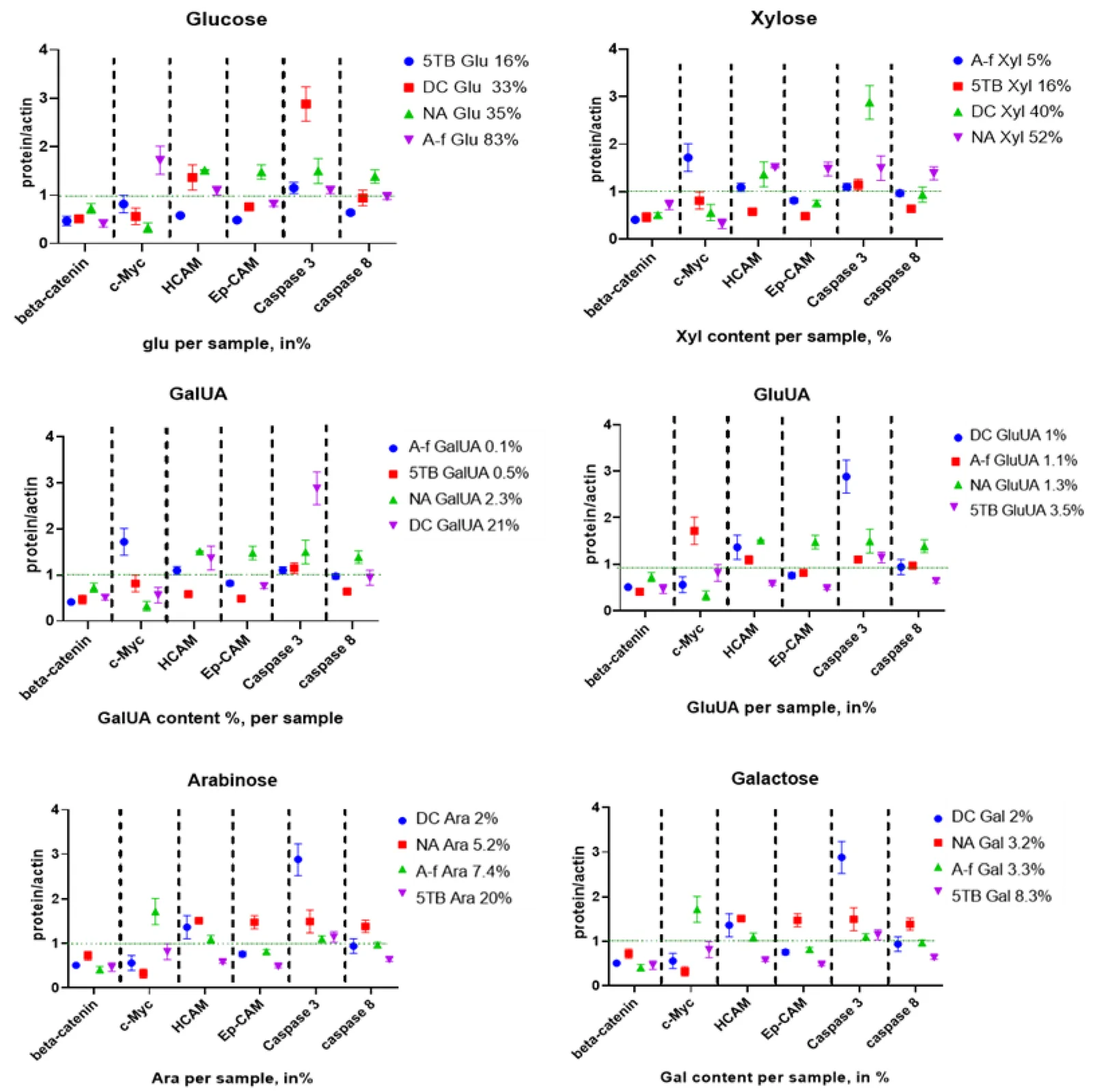
Relationship between the percentage increase in monosaccharides and protein expression levels in CSCs. (**A**) Glucose, (**B**) xylose, (**C**) GalUA, (**D**) GluUA, (**E**) arabinose, and (**F**) galactose.

To assess the biological activity of the tested samples, we examined the structure–function relationship between their monosaccharide composition and associated protein expression levels. Specifically, we aimed to identify individual monosaccharides whose concentrations—ranging from low to high—exhibited a linear correlation with protein expression. All significant variations in monosaccharide content and their corresponding protein expression levels were mapped (Figure 8). 

**Figure 9:**
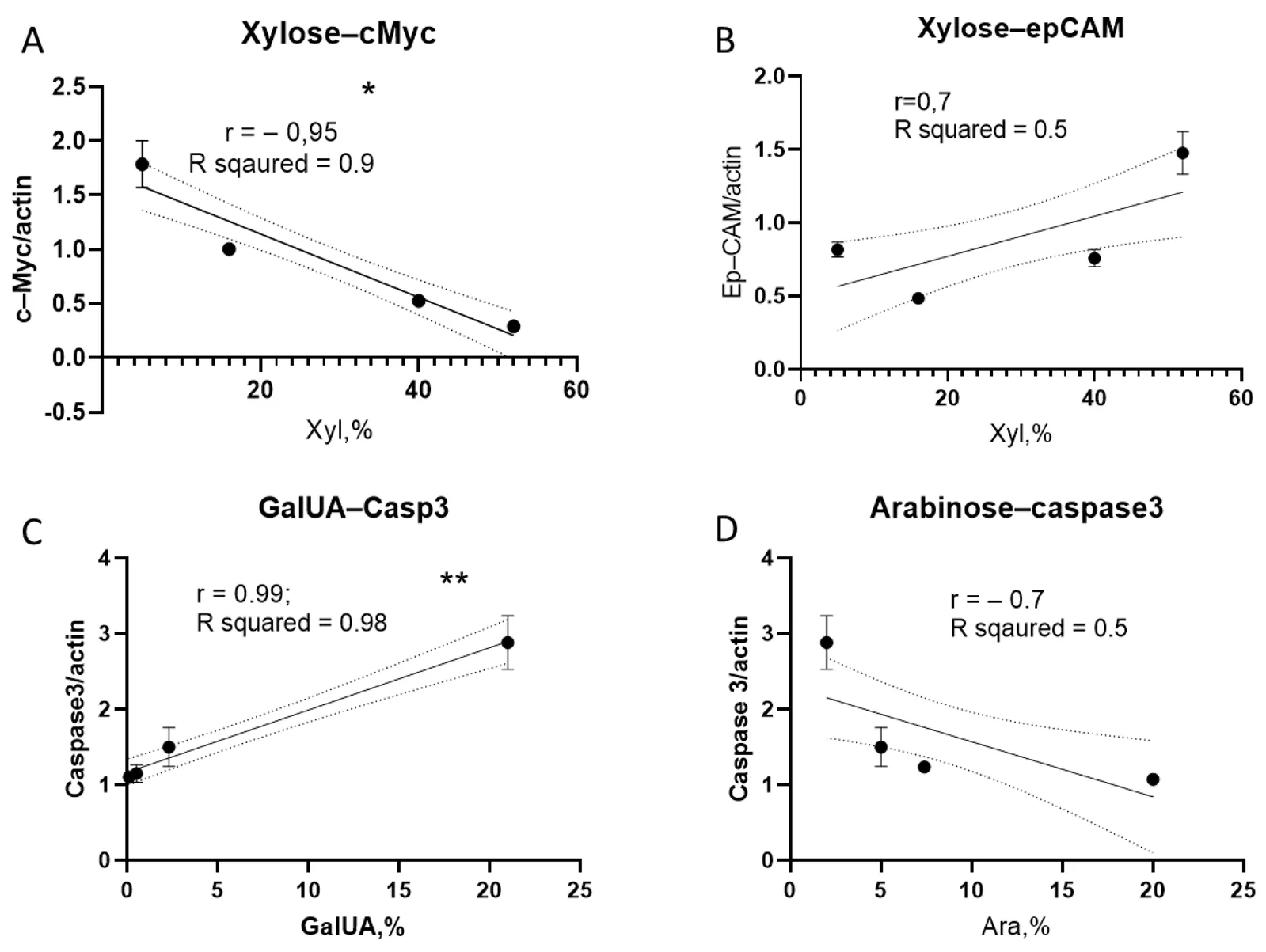
**Correlation between monosaccharide content and protein expression in CSC.** (**A**) Correlation between xylose content and c-Myc expression; (**B**) correlation between xylose content and EpCAM expression; (**C**) correlation between GalUA content and caspase-3 levels; and (**D**) correlation between arabinose content and caspase-3 levels (** *p* < 0.01; * *p* < 0.05).

No significant correlation was observed between glucose content and protein expression (Figure 9A). In contrast, xylose content showed a significant negative correlation with c-Myc protein levels (Figure 9A,B), and a non-significant positive correlation with EpCAM expression (Figure 9B). These findings suggest that increased xylose concentration in the polysaccharide samples is associated with reduced c-Myc expression.

A significant positive correlation was also found between GalUA content and caspase-3 expression levels (Figure 9C). Although a negative trend was observed between arabinose content and caspase-3 expression, this association did not reach statistical significance (Figure 9D).

**Figure 10:**
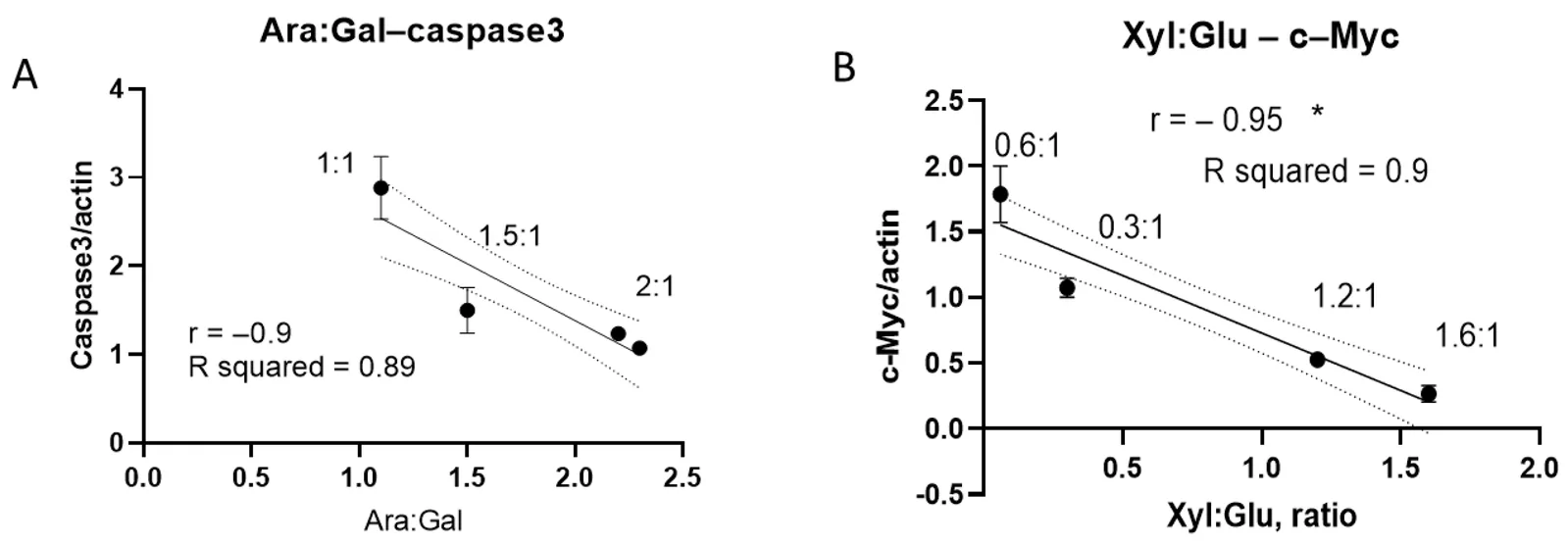
**Impact of monosaccharide ratios on protein expression in CSCs.** (**A**) Correlation between arabinose/galactose ratio and caspase-3 expression levels across different samples (1:1—DC sample; 1.5:1—NA; 2:1—A-f and 5TB); (**B**) correlation between xylose/glucose ratio and c-Myc expression (0.06:1—A-f sample; 0.3:1—5TB; 1.2:1—DC; 1.6—NA). (* *p* < 0.05).

Additionally, we explored how monosaccharide ratios might influence protein expression. A higher arabinose-to-galactose ratio was associated with a reduction in caspase-3 levels (Figure 10A). Similarly, an increased xylose-to-glucose ratio correlated negatively with c-Myc expression (Figure 10B). These results indicate that specific monosaccharide ratios, rather than absolute concentrations, may modulate the expression of key regulatory proteins.

## 4. Discussion

PSs derived from natural sources have garnered attention for their potential antitumor properties. Specifically, CSCs are known to exhibit unique glycosylation patterns that play a crucial role in proliferation, drug resistance, metastasis, and immune evasion [50,51,52]. Recent studies suggest that modulating the glycome of CSCs could represent a promising therapeutic strategy [53,54,55,56,57,58,59,60]. This study aimed to evaluate the ability of wheat cell culture PSs to target and modulate CSC populations. We hypothesized that these fractions could selectively impact CSC characteristics, including self-renewal, differentiation, and treatment resistance, potentially overcoming limitations of conventional therapies. Our findings highlight the bioactive properties of WCCPS fractions and their capacity to interfere with CSC biology, offering valuable insights into the development of CSC-targeted therapies for CRC.

Previously, we identified six WCCPS samples exhibiting anticancer effects on the differentiated HCT-116 colon cancer cell line [22]. In the present study, we tested WCCPS samples derived from liquid medium (extracellular fractions) with varying media composition and cultivation time, as well as samples extracted from suspension cells (cellular fractions). As a result, four samples showed inhibitory activity against colon CSCs derived from HCT-116 cells: extracellular PS samples—A-f (from the previous investigation) [22], 5TB, and NA; cellular PS sample: DC.

The structural diversity of PSs is largely influenced by factors such as their source, extraction method, monosaccharide content, and modification strategies [61,62]. Additionally, natural PSs often undergo structural modifications, such as acetylation, methylation, or branching, which can contribute to their bioactivity [63]. Monosaccharides, as the fundamental building blocks of PSs, serve as a simple yet crucial indicator for bioactivity assessment. Analyzing the composition and relative abundance of monosaccharides provides valuable insights into the structure–bioactivity relationship of PSs, offering a promising approach for understanding their functional properties [64]. This approach revealed significant variations in the monosaccharide composition across the different WCCPS fractions, underscoring the complexity of these molecules.

Plant cell cultivation duration significantly influences the composition and bioactivity of secreted PSs [22,65,66]. In medium 2 (ABA-containing), the A-f sample from long-term culture (1 mg/L ABA) exhibited an IC_50_ of 10 µg/mL in DCCs compared to 1181 µg/mL for the NA sample from medium-term culture (0.5 mg/L ABA), while both samples showed similar IC_50_s in CSCs (~1535 vs. ~1579 µg/mL). These differences correlate with their monosaccharide profiles: NA is enriched in xyloglucans (52% xylose, 35% glucose), whereas A-f is predominantly beta-glucan (83.2% glucose, with minor arabinose and xylose). Similarly, in medium 1 (2,4-D), the extracellular 5TB and cellular DC fractions showed distinct bioactivities, with DC exhibiting a lower IC_50_ in CSCs (832 µg/mL) compared to 5TB (1068 µg/mL), despite a higher IC_50_ in DCCs (1582 vs. 513 µg/mL). Notably, the DC fraction is enriched in galacturonic acid and xyloglucans, and given the documented anticancer properties of galacturonans [67], these structural differences likely underlie the observed variations in bioactivity.

The peculiarities in the monosaccharide composition of WCCPSs exert a significant influence on the biology of CSCs in CRC. In fact, among the tested fractions, the NA and DC samples exhibited the most pronounced inhibitory effects on CSCs. Notably, both fractions are rich in xylose, which may contribute to their bioactivity. The superior inhibition of CSCs by the NA sample, compared to DCCs, can be linked to its high xylose and glucose content. Previous studies have demonstrated that xyloglucans possess antitumor properties—for instance, xyloglucan selenious ester and sulfated xyloglucan showed activity against liver cancer cells in vitro [68], xyloglucan oligosaccharides inhibited COLO201 cell growth [69], and xyloglucan from Copaifera langsdorffii seeds impaired melanoma cell mitochondrial function [70]. Moreover, a xyloglucan and oxovanadium complex inhibited HepG2 tumor cells [71], and xylan selectively inhibited HeLa cells without affecting normal cell proliferation [72]. However, no evidence to date supports the ability of xyloglucans or xylans to inhibit CSCs. In this context, we hypothesize that the selective effect of the NA sample on CSCs may be attributed to the adhesive properties of xyloglucans, as previously reported [73,74]. Xyloglucans are mucoadhesive PSs with a high affinity for mucosal surfaces, which are rich in mucins—key mediators of tumor proliferation, progression, and therapy resistance due to their roles in regulating the epithelial-to-mesenchymal transition (EMT) and supporting CSC maintenance [75]. We therefore propose that xyloglucans in the NA fraction interact with mucins and their receptors on the CSC surface, modulating stemness-related pathways.

Furthermore, the DC fraction exhibited a more potent inhibition of CSC proliferation, as indicated by its lower IC_50_ compared to NA, and was notably enriched in GalUA. This elevated GalUA content allows classification of the DC fraction as a galacturonan [76], suggesting that its structural composition—particularly the combined presence of xylose and GalUA—underpins its enhanced bioactivity against CSCs. Given that GalUA is a principal constituent of pectins, which possess well-documented anticancer activity in colorectal cancer [77], its integration into complex polysaccharide matrices may play a pivotal functional role. CSCs, characterized by distinct receptor repertoires and a specialized tumor microenvironment [56,78], are especially responsive to pectin-derived PSs. Prior studies have implicated pectin activity in interactions with Toll-like receptor 4 and galectin-3 [79,80]. Galectin-3, a multifunctional lectin involved in cellular proliferation, aggregation, migration, survival, and apoptosis [81], has been closely linked to the maintenance of CSC stemness [82,83]. Thus, we propose that the selective inhibitory effect of the DC fraction on CSCs may be mediated through its interaction with galectin-3 and associated mucin receptors. In contrast, the effect of the NA fraction is likely driven predominantly by interactions with mucins. Together, these mechanisms may modulate critical self-renewal and tumorigenic pathways in CSCs, which are distinct from those sustaining DCCs.

Differences in monosaccharide composition may underlie the distinct effects of these samples on protein expression, as determined by Western blot analysis. Regarding medium 2-cultivated samples (A-f and NA), A-f significantly reduced β-catenin levels, increased c-Myc expression, maintained CD44 levels, and markedly decreased Ep-CAM in CSCs, suggesting inhibition of the Wnt/β-catenin pathway [84]. The concomitant rise in c-Myc may indicate a compensatory mechanism to sustain proliferation, a phenomenon frequently observed in CSC-targeted therapies and possibly due to the complex regulation by Wnt-responsive elements or aberrant signaling [85]. In contrast, the NA sample inhibited both β-catenin and c-Myc while increasing CD44 and Ep-CAM levels, implying a dual effect in supporting CSC stemness yet suppressing EMT. Additionally, NA enhanced pro-caspase-3 (*p* < 0.05) and caspase-8 (*p* < 0.01) levels, indicating activation of the extrinsic apoptotic pathway, potentially via death receptor signaling [86], although cytochrome C levels remained unchanged, possibly reflecting a preferential activation of Smac/DIABLO [87,88].

For medium 1 samples (5TB and DC), protein expression analysis revealed that 5TB significantly reduced β-catenin (*p* < 0.001), CD44 (*p* < 0.0001), and Ep-CAM (*p* < 0.0001), thereby strongly inhibiting both EMT and CSC stemness. In contrast, DC treatment resulted in significant decreases in β-catenin (*p* < 0.0001), c-Myc (*p* < 0.001), and Ep-CAM (*p* < 0.01), with no effect on CD44. Notably, DC increased pro-caspase-3 levels (*p* < 0.001), without inducing cytochrome C and caspase-8 overexpression.

While the precise mechanisms underlying CSC death induced by WCCPS fractions remain to be fully elucidated, several key trends emerged from our findings: (i) All fractions tested inhibited β-catenin expression, thereby suppressing the Wnt/β-catenin pathway, a critical axis in the epithelial-to-mesenchymal transition (EMT) and tumor progression [89,90,91,92]. Given the central role of Wnt/β-catenin signaling in CRC and CSC maintenance, these results suggest that WCCPSs may act as effective pathway inhibitors, comparable to PSs derived from *Dendrobium officinale* and *Astragalus* [93]. (ii) c-Myc levels were significantly reduced in both DC and NA samples, indicative of diminished CSC proliferative capacity [94]. Silencing c-Myc expression via siRNA in colon CSCs has been shown to reduce tumorigenicity in vivo, impair CD133⁺ cell invasion and migration in vitro, and inhibit tumor sphere formation. Moreover, c-Myc suppression may sensitize CSCs to chemotherapy-induced cytotoxicity [95], underscoring its therapeutic relevance. Given its role as a master regulator of self-renewal and chemoresistance, the c-Myc downregulation observed with DC and NA highlights their promise as CSC-targeting agents. (iii) Most WCCPS samples, except NA, decreased Ep-CAM expression, a surface marker associated with stemness and poor prognosis in CRC [96], suggesting a disruption of CSC maintenance mechanisms, (iv) The 5TB sample reduced CD44, a transmembrane glycoprotein and key stemness marker involved in EMT, invasion, metastasis, and therapy resistance. As CD44 is also a negative prognostic factor in various cancers [97], this effect reinforces the therapeutic potential of selected WCCPSs. (v) None of the fractions altered cytochrome C levels, indicating that apoptosis was not triggered via the intrinsic mitochondrial pathway [98,99]. (vi) The DC sample notably increased pro-caspase 3 expression, while NA produced a modest elevation. Although traditionally viewed as an executioner of apoptosis, caspase-3 is increasingly recognized for its non-apoptotic roles, including the promotion of stem cell differentiation [100]. The absence of cytochrome C release and caspase-8 overexpression, alongside elevated pro-caspase 3, suggests that DC does not induce apoptosis but rather facilitates CSC differentiation. This aligns with prior studies in embryonic stem cells showing upregulation of pro-caspase-3 during differentiation in the absence of early cytochrome C release [100,101]. Taken together, the DC fraction’s ability to reduce c-Myc and Ep-CAM, without triggering apoptosis, supports its role in promoting CSC differentiation. These findings reinforce the hypothesis that pro-caspase-3 may act as a critical mediator of differentiation in CSCs independent of apoptotic signaling [100]. Importantly, DC contains 21% galacturonic acid, which may be relevant to its biological activity. (vii) Finally, the 5TB sample reduced caspase-8 levels—an effect that could be detrimental, as decreased caspase-8 has been associated with enhanced cancer progression [102]. Consistently, this sample showed a concentration-dependent trend (50–100 µg/mL) toward increased CSC proliferation.

To further elucidate the differences in monosaccharide composition between the NA and DC samples, a correlation analysis between monosaccharide content and protein expression was performed [45]. The results suggest that the greater inhibitory effect of the NA sample on CSCs, compared to DCCs, may be attributed to its stronger downregulation of c-Myc protein expression. This, in turn, appears to be associated with its higher xylose content. Specifically, xylose and glucose together accounted for 86.6% of the monosaccharides in the NA sample (52% and 34.6%, respectively), compared to 73% in the DC sample (40% xylose and 34% glucose). Moreover, both the absolute xylose content and the xylose/glucose ratio showed a negative correlation with c-Myc protein levels. The NA sample exhibited a higher xylose/glucose ratio (1.6:1) than the DC sample (1.2:1), which may underlie its more pronounced inhibitory effect on c-Myc. These findings suggest that the enrichment of xyloglucans and xylans in the NA sample contributes to its ability to suppress c-Myc expression, potentially explaining its enhanced activity against CSCs relative to DCCs.

Although direct evidence for the inhibitory action of xyloglucans or xylans on CSCs is currently lacking, the observed negative correlation between xylose content and c-Myc protein expression is particularly relevant, suggesting a potential selective effect on CSC biology. Considering that c-Myc is a key regulator of self-renewal and resistance mechanisms in CSCs [94,95], its downregulation could represent a pivotal strategy to diminish stemness, especially in relation to EMT processes [103,104]. This hypothesis gains further support from studies reporting interactions between xyloglucans and mucins [74], glycoproteins implicated in the modulation of both stemness and EMT-related pathways [75]. In particular, the mucin receptor MUC1—frequently overexpressed in CSCs—has been shown to activate the Wnt/β-catenin/TCF4 signaling cascade, promote MYC transcription, and directly bind to c-Myc, thereby regulating its target genes and reinforcing the maintenance of stemness [105]. These insights suggest that xyloglucans in the NA sample may influence c-Myc expression through interactions with mucin receptors, such as MUC1, a well-established surface marker in colorectal CSCs [75,106]. The therapeutic relevance of this axis is further highlighted by the efficacy of MUC1-targeted vaccines in murine models [107], underscoring the potential of exploiting mucin-mediated mechanisms for CSC-directed therapies.

On the other hand, we observed that the high content of GalUA in the DC sample may contribute to increased caspase-3 expression, potentially explaining its stronger inhibitory effect on both CSCs and DCCs compared to the NA sample. The DC sample is characterized by notable levels of pectins, xylans, and xyloglucans, along with a relatively low arabinogalactan content. Notably, it also presents the lowest arabinose/galactose ratio (1:1), suggesting that a reduced proportion of arabinogalactans may correlate with enhanced caspase-3 activation. Thus, the combined presence of GalUA-rich pectic PSs and high xylose, alongside low arabinogalactan levels, could underlie the broader anticancer effects of the DC sample.

Previous studies have demonstrated that pectic PSs can upregulate caspase-3 in various cancer models [61,108], while the role of arabinogalactans in this context remains unclear due to limited supporting evidence. Collectively, the specific compositional features of the DC sample may explain its enhanced capacity to suppress DCCs at all concentrations and CSCs at higher doses, relative to the NA sample.

We hypothesize that the distinct monosaccharide profiles of the NA and DC samples engage different cell surface receptors, differentially expressed in DCCs and CSCs [56,109]. Although receptor profiling was beyond the scope of this study, literature reports indicate that PSs can interact with receptors such as galectin-3 and CD44—molecules frequently overexpressed in CSCs [83,110,111,112,113]. These differential interactions may underlie the cell-type-specific effects observed. Future work should focus on identifying receptor–ligand dynamics through receptor expression analyses and functional blocking experiments.

A key limitation of this study is the lack of precise structural characterization of the polysaccharide fractions. Future work will focus on the isolation and characterization of WCCPSs to better understand their biological effects, particularly their interaction with cancer-related receptors such as galectin-3, and other possible receptors. Moreover, further structural analyses will be essential to identify the specific PS components responsible for these effects. To further clarify their mechanisms of action, advanced approaches such as transcriptome profiling and receptor interaction analyses will be employed to better define the mechanisms of action of these natural compounds. Additionally, different purification methods, including enzymatic digestion and fractionation techniques, will be explored to enhance the anticancer effects of WCCPSs, as these approaches have been reported to improve the activity of pectic PSs [114].

One of the main challenges for the therapeutic application of natural PSs is their potential hydrolytic instability, particularly in the presence of hydrolases in vivo, which may reduce their bioactivity before reaching their intended cellular targets [115,116]. To overcome this limitation, nanomedicine-based strategies, such as polysaccharide encapsulation in nanoparticles, may enhance their stability and controlled release, thereby improving their therapeutic potential [116]. Interestingly, pectic PSs have been reported to possess the ability to self-assemble into nanoparticles, which could further facilitate their biomedical application [117]. Exploring these strategies in future studies will be essential for optimizing their efficacy in CRC treatment.

## 5. Conclusions

This study provides the first proof of concept demonstrating that WCCPSs possess the potential to inhibit CSCs in CRC. PSs obtained from wheat callus cell cultures exhibited distinct anti-CSC activities, with the NA and DC fractions showing the strongest effects on CSC inhibition. NA, enriched in xylose, was effective at lower concentrations, while DC, which contains both xylose and GalUA, displayed superior potency, with a significantly lower IC_50_ and greater selectivity for CSCs at higher doses. Correlation analysis revealed that xylose content was associated with reduced c-Myc expression, while GalUA levels were linked to increased caspase-3 activation. These findings support the hypothesis that the biological activity of WCCPSs is modulated by their monosaccharide composition. Collectively, our results highlight the potential of wheat-derived PSs as agents that target CSCs, suppressing their self-renewal and promoting differentiation, thereby offering a promising strategy to reduce tumor aggressiveness and recurrence. Further research should focus on elucidating the molecular mechanisms underlying these effects, particularly the involvement of the Wnt/β-catenin pathway and the specific polysaccharide components responsible for these activities. Additionally, in vivo studies are necessary to evaluate the therapeutic potential and safety of WCCPSs, with nanotechnology-based delivery systems possibly enhancing their bioactivity and stability for clinical applications.

## Figures and Tables

**Table 1:** Percent of monosaccharides in WCCPS samples.

	NA	A-f	DC	5TB
Ara	5.2	7.4	2	19.7
Xyl	52	4.8	40	15.9
Gal	3.2	3.3	2	8.3
Glu	34.6	83.2	33	52.2
GlcUA	1.3	1.1	1	3.5
GalUA	2.3	0.1	21	0.5
Man	1.3	0	1	0

## Data Availability

The original contributions presented in this study are included in the article. Further inquiries can be directed to the corresponding authors.

## Abbreviations

WCCPSs	Wheat cell culture polysaccharides
CSCs	Cancer stem cells
DCCs	Differentiated cancer cells
CRC	Colorectal cancer
EMT	Endothelial–mesenchymal transition
PSs	Polysaccharides

## References

[1] World Health Organization. https://www.who.int/news-room/fact-sheets/detail/colorectal-cancer.

[2] Kozovska Z., Gabrisova V., Kucerova L. (2014). Colon cancer: Cancer stem cells markers, drug resistance and treatment. Biomed. Pharmacother..

[3] Housman G., Byler S., Heerboth S., Lapinska K., Longacre M., Snyder N., Sarkar S. (2014). Drug Resistance in Cancer: An Overview. Cancers.

[4] Zhou Y., Xia L., Wang H., Oyang L., Su M., Liu Q., Lin J., Tan S., Tian Y., Liao Q. (2018). Cancer stem cells in progression of colorectal cancer. Oncotarget.

[5] Loh J.J., Ma S. (2024). Hallmarks of cancer stemness. Cell Stem Cell.

[6] Hanahan D. (2022). Hallmarks of Cancer: New Dimensions. Cancer Discov..

[7] Liu Z., Xu H., Weng S., Ren Y., Han X. (2022). Stemness Refines the Classification of Colorectal Cancer with Stratified Prognosis, Multi-Omics Landscape, Potential Mechanisms, and Treatment Options. Front. Immunol..

[8] Dadgar T., Ebrahimi N., Gholipour A.R., Akbari M., Khani L., Ahmadi A., Hamblin M.R. (2022). Targeting the metabolism of cancer stem cells by energy disruptor molecules. Crit. Rev. Oncol./Hematol..

[9] Asma S.T., Acaroz U., Imre K., Morar A., Shah S.R.A., Hussain S.Z., Arslan-Acaroz D., Demirbas H., Hajrulai-Musliu Z., Istanbullugil F.R. (2022). Natural Products/Bioactive Compounds as a Source of Anticancer Drugs. Cancers.

[10] Moselhy J., Srinivasan S., Ankem M.K., Damodaran C. (2015). Natural Products That Target Cancer Stem Cells. Anticancer. Res..

[11] Sung C.-J., Wang H.-H., Sun K.-H., Hsieh C.-C., Huang R., Sun G.-H., Tang S.-J. (2022). Fucoidan from Sargassum hemiphyllum inhibits the stemness of cancer stem cells and epithelial-mesenchymal transitions in bladder cancer cells. Int. J. Biol. Macromol..

[12] Tacchini M., Sacchetti G., Guerrini A., Paganetto G. (2023). Mycochemicals against Cancer Stem Cells. Toxins.

[13] Hermawan A., Putri H. (2018). Current report of natural product development against breast cancer stem cells. Int. J. Biochem. Cell Biol..

[14] Gupta P.K., Saraff M., Gahtori R., Negi N., Tripathi S.K., Kumar J., Kumar S., Aldhayan S.H., Dhanasekaran S., Abomughaid M.M. (2021). Phytomedicines Targeting Cancer Stem Cells: Therapeutic Opportunities and Prospects for Pharmaceutical Development. Pharmaceuticals.

[15] Guo R., Chen M., Ding Y., Yang P., Wang M., Zhang H., He Y., Ma H. (2022). Polysaccharides as Potential Anti-tumor Biomacromolecules -A Review. Front. Nutr..

[16] Fan J., Zhu J., Zhu H., Zhang Y., Xu H. (2024). Potential therapeutic target for polysaccharide inhibition of colon cancer progression. Front. Med..

[17] Pang G., Wang F., Zhang L.W. (2018). Dose matters: Direct killing or immunoregulatory effects of natural polysaccharides in cancer treatment. Carbohydr. Polym..

[18] Meng X., Liang H., Luo L. (2016). Antitumor polysaccharides from mushrooms: A review on the structural characteristics, antitumormechanisms and immunomodulating activities. Carbohydr. Res..

[19] Liu M.-M., Zeng P., Li X.-T., Shi L.-G. (2016). Antitumor and immunomodulation activities of polysaccharide from *Phellinus baumii*. Int. J. Biol. Macromol..

[20] Ramawat K.G., Merillon J.-M. (2015). Polysaccharides Bioactivity and Biotechnology.

[21] Ju H., Yu C., Zhang X.-D., Liu W., Wu Y.-C., Gong P.-X. (2023). Recent trends in anti-cancer activities of terrestrial plants-based polysaccharides: A review. Carbohydr. Polym. Technol. Appl..

[22] Murtazina A., Ruiz Alcala G., Jimenez-Martinez Y., Marchal J.A., Tarabayeva A., Bitanova E., McDougall G., Bishimbayeva N., Boulaiz H. (2022). Anti-Cancerous Potential of Polysaccharides Derived from Wheat Cell Culture. Pharmaceutics.

[23] de Camargo M.R., Frazon T.F., Inacio K.K., Smiderle F.R., Amôr N.G., Dionísio T.J., Santos C.F., Rodini C.O., Lara V.S. (2022). Ganoderma lucidum polysaccharides inhibit *in vitro* tumorigenesis, cancer stem cell properties and epithelial-mesenchymal transition in oral squamous cell carcinoma. J. Ethnopharmacol..

[24] Hu B., Yan W., Wang M., Cui X., Hu Y., Chen Q., Zhang Y., Qi X., Jiang J. (2019). Huaier polysaccharide inhibits the stem-like characteristics of ERα-36high triple negative breast cancer cells via inactivation of the ERα-36 signaling pathway. Int. J. Biol. Sci..

[25] Hafez H.G., Mohareb R.M., Salem S.M., Matloub A.A., Eskander E.F., Ahmed H.H. (2022). Molecular Mechanisms Underlying the Anti-Breast Cancer Stem Cell Activity of Pterocladia capillacea and Corallina officinalis Polysaccharides. Anti-Cancer Agents Med. Chem..

[26] Cotas J., Marques V., Afonso M.B., Rodrigues C.M.P., Pereira L. (2020). Antitumour Potential of Gigartina pistillata Carrageenans against Colorectal Cancer Stem Cell-Enriched Tumourspheres. Mar. Drugs.

[27] Ai F., Xiao H., Wang F., Zhu Y., Ma L. (2024). Reversal effect of Lycium barbarum polysaccharide in combination with oxaliplatin on drug resistance of colon cancer stem cells. Chin. J. Tissue Eng. Res..

[28] Peng Q., Yu Y., Ye L., Zhang S., Li Y., Hua X., Shen S., Hu D., Lu W. (2024). Astragalus polysaccharides sensitize ovarian cancer stem cells to PARPi by inhibiting mitophagy via PINK1/Parkin signaling. iScience.

[29] Chang P.H., Sekine K., Chao H.M., Hsu S.H., Chern E. (2017). Chitosan promotes cancer progression and stem cell properties in association with Wnt signaling in colon and hepatocellular carcinoma cells. Sci. Rep..

[30] Murashige T., Skoog F. (1962). A revised medium for rapid growth and bioassays with tobacco tissue cultures. Phisiol. Plant..

[31] Bishimbayeva N.K., Murtazina A.S., McDougall G.J. (2017). Influence of Phytohormones on Monosaccharide Composition of Polysaccharides from Wheat Suspension Culture. Eurasian Chem. J..

[32] Parmar S.S., Sainger M., Chaudhary D., Jaiwal P.K. (2012). Plant regeneration from mature embryo of commercial Indian bread wheat (*Triticum aestivum* L.) cultivars. Physiol. Mol. Biol. Plants.

[33] Mehaboob V.M., Faizal K., Raja P., Thiagu G., Aslam A., Shajahan A. (2019). Effect of nitrogen sources and 2, 4-D treatment on indirect regeneration of ginger (*Zingiber officinale* Rosc.) using leaf base explants. J. Plant Biotechnol..

[34] Karimian R., Lahouti M., Davarpanah S.J. (2014). Effects of Different Concentrations of 2, 4-D and Kinetin on Callogenesis of Taxus Brevifolia Nutt. J. Appl. Biotechnol. Rep..

[35] Markowski M., Alsoufi A.S.M., Szakiel A., Długosz M. (2022). Effect of Ethylene and Abscisic Acid on Steroid and Triterpenoid Synthesis in Calendula officinalis Hairy Roots and Saponin Release to the Culture Medium. Plants.

[36] Bishimbayeva N.K., Sartbayeva I.A., Murtazina A.S., Günter E.A. (2015). Chemical Composition of Polysaccharides from Wheat Cell Culture. Int. J. Biol. Chem..

[37] Gunter E.A. (2001). Polysaccharides from callus culture *Lemna minor* L.. Chem. Plants Raw Mater..

[38] Günter E.A., Ovodov Y.S. (2002). Changes in cell wall polysaccharides of Silene vulgaris callus during culture. Phytochemistry.

[39] Dubois M., Gilles K.A., Hamilton J.K., Rebers P.A., Smith F. (1956). Colometric Method for Determination of Sugars and Related Substances. Anal. Chem..

[40] Ross H.A., Wright K.M., McDougall G.J., Roberts A.G., Chapman S.N., Morris W.L., Hancock R.D., Stewart D., Tucker G.A., James E.K. (2011). Potato tuber pectin structure is influenced by pectin methyl esterase activity and impacts on cooked potato texture. J. Exp. Bot..

[41] Morata-Tarifa C., Jiménez G., García M., Entrena J.M., Griñán-Lisón C., Aguilera M., Picon-Ruiz M., Marchal J.A. (2016). Low adherent cancer cell subpopulations are enriched in tumorigenic and metastatic epithelial-to-mesenchymal transition-induced cancer stem-like cells. Sci. Rep..

[42] Palacios-Ferrer J.L., García-Ortega M.B., Gallardo-Gómez M., García M.Á., Díaz C., Boulaiz H., Valdivia J., Jurado J.M., Almazan-Fernandez F.M., Arias-Santiago S. (2021). Metabolomic profile of cancer stem cell-derived exosomes from patients with malignant melanoma. Mol. Oncol..

[43] Ramírez A., Boulaiz H., Tarifa C.M., Perán M., Jiménez G., Ruiz M.P., Agil A., Cruz-López O., Conejo-García A., Campos J.M. (2014). HER2-signaling pathway, JNK and ERKs kinases, and cancer stem-like cells are targets of Bozepinib. Oncotarget.

[44] Cáceres B., Ramirez A., Carrillo E., Jimenez G., Griñán-Lisón C., López-Ruiz E., Jiménez-Martínez Y., Marchal J.A., Boulaiz H. (2019). Deciphering the Mechanism of Action Involved in Enhanced Suicide Gene Colon Cancer Cell Killer Effect Mediated by Gef and Apoptin. Cancers.

[45] Han P., Yao S., Guo R., Yan R., Wu Y., Shen S., Jia S. (2017). Influence of culture conditions on extracellular polysaccharide production and the activities of enzymes involved in the polysaccharide synthesis of Nostoc flagelliforme. RSC Adv..

[46] Fattore L., Mancini R., Ciliberto G. (2020). Cancer Stem Cells and the Slow Cycling Phenotype: How to Cut the Gordian Knot Driving Resistance to Therapy in Melanoma. Cancers.

[47] Moore N., Lyle S. (2011). Quiescent, slow-cycling stem cell populations in cancer: A review of the evidence and discussion of significance. J. Oncol..

[48] Franken N., Rodermond H., Stap J., Haveman J., van Bree C. (2006). Clonogenic assay of cells *in vitro*. Nat. Protoc..

[49] Brix N., Samaga D., Hennel R., Gehr K., Zitzelsberger H., Lauber K. (2020). The clonogenic assay: Robustness of plating efficiency-based analysis is strongly compromised by cellular cooperation. Radiat. Oncol..

[50] Nardy A.F., Freire-de-Lima L., Freire-de-Lima C.G., Morrot A. (2016). The Sweet Side of Immune Evasion: Role of Glycans in the Mechanisms of Cancer Progression. Front. Oncol..

[51] Very N., Lefebvre T., El Yazidi-Belkoura I. (2018). Drug resistance related to aberrant glycosylation in colorectal cancer. Oncotarget.

[52] Taniguchi N., Kizuka Y. (2015). Glycans and cancer: Role of N-glycans in cancer biomarker, progression and metastasis, and therapeutics. Adv. Cancer Res..

[53] Dennis J.W., Nabi I.R., Demetriou M. (2009). Metabolism, cell surface organization, and disease. Cell.

[54] Carvalho-Cruz P., Alisson-Silva F., Todeschini A.R., Dias W.B. (2018). Cellular glycosylation senses metabolic changes and modulates cell plasticity during epithelial to mesenchymal transition. Dev. Dyn..

[55] Scheper A.F., Schofield J., Bohara R., Ritter T., Pandit A. (2023). Understanding glycosylation: Regulation through the metabolic flux of precursor pathways. Biotechnol. Adv..

[56] Khan T., Cabral H. (2021). Abnormal Glycosylation of Cancer Stem Cells and Targeting Strategies. Front. Oncol..

[57] Walker M.R., Goel H.L., Mukhopadhyay D., Chhoy P., Karner E.R., Clark J.L., Liu H., Li R., Zhu J.L., Chen S. (2022). O-linked α2,3 sialylation defines stem cell populations in breast cancer. Sci. Adv..

[58] Schultz M.J., Holdbrooks A.T., Chakraborty A., Grizzle W.E., Landen C.N., Buchsbaum D.J., Conner M.G., Arend R.C., Yoon K.J., Klug C.A. (2016). The Tumor-Associated Glycosyltransferase ST6Gal-I Regulates Stem Cell Transcription Factors and Confers a Cancer Stem Cell Phenotype. Cancer Res..

[59] Buffone A., Weaver V.M. (2020). Don’t sugarcoat it: How glycocalyx composition influences cancer progression. J. Cell Biol..

[60] Vitale D., Kumar Katakam S., Greve B., Jang B., Oh E.-S., Alaniz L., Götte M. (2019). Proteoglycans and glycosaminoglycans as regulators of cancer stem cell function and therapeutic resistance. FEBS J..

[61] Maksymowicz J., Palko-Łabuz A., Sobieszczańska B., Chmielarz M., Ferens-Sieczkowska M., Skonieczna M., Wikiera A., Wesołowska O., Środa-Pomianek K. (2022). The Use of Endo-Cellulase and Endo-Xylanase for the Extraction of Apple Pectins as Factors Modifying Their Anticancer Properties and Affecting Their Synergy with the Active Form of Irinotecan. Pharmaceuticals.

[62] Benalaya I., Alves G., Lopes J., Silva L.R. (2024). A Review of Natural Polysaccharides: Sources, Characteristics, Properties, Food, and Pharmaceutical Applications. Int. J. Mol. Sci..

[63] Singh N.K., Baranwal J., Pati S., Barse B., Khan R.H., Kumar A. (2023). Application of plant products in the synthesis and functionalisation of biopolymers. Int. J. Biol. Macromol..

[64] Wang Z., Zheng Y., Lai Z., Hu X., Wang L., Wang X., Li Z., Gao M., Yang Y., Wang Q. (2024). Effect of monosaccharide composition and proportion on the bioactivity of polysaccharides: A review. Int. J. Biol. Macromol..

[65] Efferth T. (2019). Biotechnology Applications of Plant Callus Cultures. Engineering.

[66] Deshpande A., Dhadi S.R., Hager E.J., Ramakrishna W. (2012). Anticancer Activity of Rice Callus Suspension Culture. Phytother. Res..

[67] Hosseini Abari A., Amini Rourani H., Ghasemi S.M., Kim H., Kim Y.G. (2021). Investigation of antioxidant and anticancer activities of unsaturated oligo-galacturonic acids produced by pectinase of Streptomyces hydrogenans YAM1. Sci. Rep..

[68] Cao Y., Ikeda I. (2009). Antioxidant activity and antitumor activity (*in vitro*) of xyloglucan selenious ester and surfated xyloglucan. Int. J. Biol. Macromol..

[69] Kato Y., Uchida J., Ito S., Mitsuishi Y. (2009). Structural analysis of the oligosaccharides units of xyloglucan and their effects on growth of COLO 201 human tumor cells. Intern. Cong. Ser..

[70] Farias C.L.A., Martinez G.R., Cadena S.M.S.C., Mercê A.L.R., de Oliveira Petkowicz C.L., Noleto G.R. (2019). Cytotoxicity of xyloglucan from *Copaifera langsdorffii* and its complex with oxovanadium (IV/V) on B16F10 cells. Int. J. Biol. Macromol..

[71] Escaliante L.A.D.S., Busato B., de Oliveira Petkowicz C.L., Cadena S.M.S.C., Noleto G.R. (2021). Cytotoxic effect of xyloglucan and oxovanadium (IV/V) xyloglucan complex in HepG2 cells. Int. J. Biol. Macromol..

[72] Melo-Silveira R.F., Fidelis G.P., Costa M.S., Telles C.B., Dantas-Santos N., de Oliveira Elias S., Ribeiro V.B., Barth A.L., Macedo A.J., Leite E.L. (2012). In vitro antioxidant, anticoagulant and antimicrobial activity and in inhibition of cancer cell proliferation by xylan extracted from corn cobs. Int. J. Mol. Sci..

[73] Piqué N., Gómez-Guillén M.D.C., Montero M.P. (2018). Xyloglucan, a Plant Polymer with Barrier Protective Properties over the Mucous Membranes: An Overview. Int. J. Mol. Sci..

[74] Esquena-Moret J. (2022). A Review of Xyloglucan: Self-Aggregation, Hydrogel Formation, Mucoadhesion and Uses in Medical Devices. Macromol.

[75] Marimuthu S., Rauth S., Ganguly K., Zhang C., Lakshmanan I., Batra S.K., Ponnusamy M.P. (2021). Mucins reprogram stemness, metabolism and promote chemoresistance during cancer progression. Cancer Metastasis Rev..

[76] Minzanova S.T., Mironov V.F., Arkhipova D.M., Khabibullina A.V., Mironova L.G., Zakirova Y.M., Milyukov V.A. (2018). Biological Activity and Pharmacological Application of Pectic Polysaccharides: A Review. Polymers.

[77] Ornelas A.C., Ferguson S., DePlaza M., Adekunle T., Basha R. (2022). Anti-Cancer Pectins and Their Role in Colorectal Cancer Treatment. Onco Ther..

[78] Aponte P.M., Caicedo A. (2017). Stemness in Cancer: Stem Cells, Cancer Stem Cells, and Their Microenvironment. Stem Cells Int..

[79] Ishisono K., Yabe T., Kitaguchi K. (2017). Citrus pectin attenuates endotoxin shock via suppression of Toll-like receptor signaling in Peyer’s patch myeloid cells. J. Nutr. Biochem..

[80] Leclere L., van Cutsem P., Michiels C. (2013). Anti-Cancer activities of pH- or heat-modified pectin. Front. Pharmacol..

[81] Nangia-Makker P., Hogan V., Raz A. (2018). Galectin-3 and cancer stemness. Glycobiology.

[82] Caputo S., Grioni M., Brambillasca C.S., Monno A., Brevi A., Freschi M., Piras I.S., Elia A.R., Pieri V., Baccega T. (2020). Galectin-3 in Prostate Cancer Stem-Like Cells Is Immunosuppressive and Drives Early Metastasis. Front. Immunol..

[83] Ilmer M., Mazurek N., Byrd J.C., Ramirez K., Hafley M., Alt E., Vykoukal J., Bresalier R.S. (2016). Cell surface galectin-3 defines a subset of chemoresistant gastrointestinal tumor-initiating cancer cells with heightened stem cell characteristics. Cell Death Dis..

[84] Wang M.H., Sun R., Zhou X.M., Zhang M.Y., Lu J.B., Yang Y., Zeng L.S., Yang X.Z., Shi L., Xiao R.W. (2018). Epithelial cell adhesion molecule overexpression regulates epithelial-mesenchymal transition, stemness and metastasis of nasopharyngeal carcinoma cells via the PTEN/AKT/mTOR pathway. Cell Death.

[85] Rennoll S., Yochum G. (2015). Regulation of MYC gene expression by aberrant Wnt/β-catenin signaling in colorectal cancer. World J. Biol. Chem..

[86] McIlwain D.R., Berger T., Mak T.W. (2013). Caspase functions in cell death and disease. Cold Spring Harb. Perspect. Biol..

[87] Arnt C., Kaufmann S. (2003). The saintly side of Smac/DIABLO: Giving anticancer drug-induced apoptosis a boost. Cell Death Differ..

[88] Zhuang S., Lynch M.C., Kochevar I.E. (1999). Caspase-8 mediates caspase-3 activation and cytochrome c release during singlet oxygen-induced apoptosis of HL-60 cells. Exp. Cell Res..

[89] Xue W., Yang L., Chen C., Ashrafizadeh M., Tian Y., Sun R. (2024). Wnt/β-catenin-driven EMT regulation in human cancers. Cell Mol. Life Sci..

[90] Sun L., Xing J., Zhou X., Song X., Gao S. (2024). Wnt/β-catenin signalling, epithelial-mesenchymal transition and crosslink signalling in colorectal cancer cells. Biomed. Pharmacother..

[91] Li Z., Yang Z., Liu W., Zhu W., Yin L., Han Z., Xian Y., Wen J., Tang H., Lin X. (2023). Disheveled3 enhanced EMT and cancer stem-like cells properties via Wnt/β-catenin/c-Myc/SOX2 pathway in colorectal cancer. J. Transl. Med..

[92] Bu H., Liu D., Cui J., Cai K., Shen F. (2019). Wnt/β-catenin signaling pathway is involved in induction of apoptosis by oridonin in colon cancer COLO205 cells. Transl. Cancer Res..

[93] Zhao H., Ming T., Tang S., Ren S., Yang H., Liu M., Tao Q., Xu H. (2022). Wnt signaling in colorectal cancer: Pathogenic role and therapeutic target. Mol. Cancer..

[94] He T.C., Sparks A.B., Rago C., Hermeking H., Zawel L., da Costa L.T., Morin P.J., Vogelstein B., Kinzler K.W. (1998). Identification of c-MYC as a target of the APC pathway. Science.

[95] Zhang H.L., Wang P., Lu M.Z., Zhang S.D., Zheng L. (2019). c-Myc maintains the self-renewal and chemoresistance properties of colon cancer stem cells. Oncol. Lett..

[96] Liu Y., Wang Y., Sun S., Chen Z., Xiang S., Ding Z., Huang Z., Zhang B. (2022). Understanding the versatile roles and applications of EpCAM in cancers: From bench to bedside. Exp. Hematol. Oncol..

[97] Xu H., Niu M., Yuan X., Wu K., Liu A. (2020). CD44 as a tumor biomarker and therapeutic target. Exp. Hematol. Oncol..

[98] Vempati U.D., Diaz F., Barrientos A., Narisawa S., Mian A.M., Millán J.L., Boise L.H., Moraes C.T. (2007). Role of cytochrome C in apoptosis: Increased sensitivity to tumor necrosis factor alpha is associated with respiratory defects but not with lack of cytochrome C release. Mol. Cell Biol..

[99] Mustafa M., Ahmad R., Tantry I.Q., Ahmad W., Siddiqui S., Alam M., Abbas K., Moinuddin, Hassan M.I., Habib S. (2024). Apoptosis: A Comprehensive Overview of Signaling Pathways, Morphological Changes, and Physiological Significance and Therapeutic Implications. Cells.

[100] Abdul-Ghani M., Megeney L.A. (2008). Rehabilitation of a contract killer: Caspase-3 directs stem cell differentiation. Cell Stem Cell.

[101] Akbari-Birgani S., Hosseinkhani S., Mollamohamadi S., Baharvand H. (2014). Delay in apoptosome formation attenuates apoptosis in mouse embryonic stem cell differentiation. J. Biol. Chem..

[102] Fianco G., Mongiardi M.P., Levi A., De Luca T., Desideri M., Trisciuoglio D., Del Bufalo D., Cinà I., Di Benedetto A., Mottolese M. (2017). Caspase-8 contributes to angiogenesis and chemotherapy resistance in glioblastoma. eLife.

[103] Liu M., Casimiro M.C., Wang C., Shirley L.A., Jiao X., Katiyar S., Ju X., Li Z., Yu Z., Zhou J. (2009). p21CIP1 attenuates Ras- and c-Myc-dependent breast tumor epithelial mesenchymal transition and cancer stem cell-like gene expression in vivo. Proc. Natl. Acad. Sci. USA.

[104] Yang J., Wu S.P., Wang W.J., Jin Z.R., Miao X.B., Wu Y., Gou D.M., Liu Q.Z., Yao K.T. (2020). A novel miR-200c/c-myc negative regulatory feedback loop is essential to the EMT process, CSC biology and drug sensitivity in nasopharyngeal cancer. Exp. Cell Res..

[105] Li W., Zhang N., Jin C., Long M.D., Rajabi H., Yasumizu Y., Fushimi A., Yamashita N., Hagiwara M., Zheng R. (2020). MUC1-C drives stemness in progression of colitis to colorectal cancer. JCI Insight.

[106] Barkeer S., Chugh S., Batra S.K., Ponnusamy M.P. (2018). Glycosylation of Cancer Stem Cells: Function in Stemness, Tumorigenesis, and Metastasis. Neoplasia.

[107] Guo M., Luo B., Pan M., Li M., Xu H., Zhao F., Dou J. (2020). Colorectal cancer stem cell vaccine with high expression of MUC1 serves as a novel prophylactic vaccine for colorectal cancer. Int. Immunopharmacol..

[108] Picot-Allain M.C.N., Neergheen V.S. (2023). Pectin a multifaceted biopolymer in the management of cancer: A review. Heliyon.

[109] Freitas C.M.P., Coimbra J.S.R., Souza V.G.L., Sousa R.C.S. (2021). Structure and Applications of Pectin in Food, Biomedical, and Pharmaceutical Industry: A Review. Coatings.

[110] Oršolić N., Jazvinšćak Jembrek M. (2024). Potential Strategies for Overcoming Drug Resistance Pathways Using Propolis and Its Polyphenolic/Flavonoid Compounds in Combination with Chemotherapy and Radiotherapy. Nutrients.

[111] Maxwell E.G., Colquhoun I.J., Chau H.K., Hotchkiss A.T., Waldron K.W., Morris V.J., Belshaw N.J. (2015). Rhamnogalacturonan I containing homogalacturonan inhibits colon cancer cell proliferation by decreasing ICAM1 expression. Carbohydr. Polym..

[112] Emran T.B., Islam F., Mitra S., Paul S., Nath N., Khan Z., Das R., Chandran D., Sharma R., Lima C.M.G. (2022). Pectin: A Bioactive Food Polysaccharide with Cancer Preventive Potential. Molecules.

[113] Jiang X.N., Dang Y.F., Gong F.L., Guo X.L. (2019). Role and regulation mechanism of Gal-3 in non-small cell lung cancer and its potential clinical therapeutic significance. Chem. Biol. Interact..

[114] Su L., Feng Y., Wei K., Xu X., Liu R., Chen G. (2021). Carbohydrate-Based Macromolecular Biomaterials. Chem. Rev..

[115] Olawuyi I.F., Park J.J., Park G.D., Lee W.Y. (2022). Enzymatic Hydrolysis Modifies Emulsifying Properties of Okra Pectin. Foods.

[116] Ginghină O., Hudiță A., Zaharia C., Tsatsakis A., Mezhuev Y., Costache M., Gălățeanu B. (2021). Current Landscape in Organic Nanosized Materials Advances for Improved Management of Colorectal Cancer Patients. Materials.

[117] Tang S., Wang T., Jiang M., Huang C., Lai C., Fan Y., Yong Q. (2019). Construction of arabinogalactans/selenium nanoparticles composites for enhancement of the antitumor activity. Int. J. Biol. Macromol..

